# Geographic and *Orientia* infection status influence on the bacterial microbiome of free-living chiggers in North Carolina, USA

**DOI:** 10.1371/journal.pone.0353174

**Published:** 2026-07-08

**Authors:** Kaiying Chen, Nicholas V. Travanty, Reuben A. Garshong, Gideon Wasserberg, Charles S. Apperson, R. Michael Roe, Loganathan Ponnusamy

**Affiliations:** 1 Department of Entomology and Plant Pathology, Comparative Medicine Institute, North Carolina State University, Raleigh, North Carolina, United States of America; 2 Department of Biology, University of North Carolina at Greensboro, Greensboro, North Carolina, United States of America; Huadong Research Institute for Medicine and Biotechniques, CHINA

## Abstract

Chiggers (larval Trombiculid mites) serve as vectors for *Orientia* species that cause scrub typhus, a potentially serious illness in humans with a broadening global distribution. To date, there is limited research on the chigger microbiome in the United States (US) compared to some other parts of the world. Investigating chigger bacterial communities is essential for understanding the potential role they play in pathogen transmission dynamics within these arthropods. This study investigated the bacterial communities of free-living chiggers collected from sites across the three ecoregions in North Carolina using 16S rDNA gene targeted next-generation sequencing. Molecular identification of the chigger revealed three species: *Eutrombicula splendens*, *Eutrombicula tinami*, and *Pseudoschoengastia* sp. All three trombiculid mite species occurred at least once in the Mountains and Piedmont, except for *E. tinami*, which was absent from the Coastal Plain ecoregion. Microbiome analysis revealed significant differences in alpha and beta diversity among the collection sites for *E. splendens*. No significant differences in overall microbiome diversity were observed between *E. splendens* and *Pseudoschoengastia* sp., the two dominant chigger species. However, the microbiome of *E. splendens* alone exhibited significant differences in both Shannon diversity and beta diversity between *Orientia*-infected and uninfected individuals. Within *E. splendens*, genera like *Brevibacillus* and *Telluria* were more abundant in *Orientia*-positive chiggers, while *Methylobacterium* was more abundant in *Orientia*-negative chiggers. We also found potentially pathogenic bacterial genera, including *Rickettsia*, *Listeria*, *Legionella*, *Staphylococcus*, and *Streptococcus* sequences. These findings suggest that geography and *Orientia* infection influence chigger-associated bacterial communities, potentially affecting their vector competence.

## Introduction

Chiggers are the ectoparasitic larval stage of mites in the families Trombiculidae and Leeuwenhoekiidae [1] that feed predominantly on terrestrial vertebrates, particularly rodents [2,3]. Worldwide, there are over 3,000 known species of trombiculid mites. Although *Orientia* has been detected in about 50 of these mite species, only about 10–15 are confirmed competent vectors of *Orientia* [4–9]. Chiggers typically feed on lymph and skin cells of their host. Their accessory chemicals and/or structures they make when feeding (the stylostome) can cause a type of dermatitis known as trombiculiasis [10], a condition which can affect both humans and animals. Chiggers, like some other arthropods, can be infected with different pathogens. Chiggers harboring *Orientia* are capable of transmitting the pathogen to humans and other animals during feeding, resulting in scrub typhus. Scrub typhus is a widespread disease, with an estimated one billion people at risk and approximately one million cases reported annually [11]. The median mortality rate for treated scrub typhus patients is approximately 1.4%; however, in untreated individuals, mortality can range from 6% to as high as 40%, depending on geographic location and the pathogenic strain involved [12–15]. Factors that affect chigger distribution, such as climate, geography, and changes in human land use, play a role in the global distribution and abundance of scrub typhus. Historically, scrub typhus was only reported in the region known as the Tsutsugamushi Triangle, which encompasses areas from Pakistan to eastern Russia to northern Australia [16]. Initially, *Orientia tsutsugamushi* was the only known causative agent of the disease. However, cases of scrub typhus caused by *Orientia chuto* were reported from the United Arab Emirates in 2006 [17], and *Candidatus* Orientia chiloensis was identified on Chiloé Island in southern Chile in 2020 [18]. The discovery that the two recently described *Orientia* species form distinct clades from *O. tsutsugamushi* and are found in regions outside its known range suggests that scrub typhus may be more geographically widespread and caused by a broader variety of *Orientia* species than previously recognized.

In addition to *Orientia*, the detection of other zoonotic microbes in chiggers has increased. Several studies have identified *Rickettsia* species known to infect humans, such as *R. japonica*, *R. akari*, *R. felis*, *R. conorii*, *R. typhi*, and other closely related species, in chiggers collected from wild rodents in Korea [19] and Taiwan [20]. The *Candidatus* Rickettsia colombianensi and *R. felis* were detected in chiggers collected from rodents and birds in Brazil [21,22]. More recently, in the United States, our group reported, for the first time, the presence of *R. felis* and other spotted fever group (SFG) *Rickettsia* spp. in chiggers collected from wild rodents [23]. Aside from pathogenic bacteria, hantavirus-specific RNA (Bayou strain) has also been detected in both rodent-associated and free-living trombiculid mites in Texas, USA [24]. The factors shaping the chigger microbiome, and thus the presence or absence of specific microbes, may include host biology, ecological interactions, and abiotic environmental conditions.

The microbiomes of organisms are essential to their functioning [25] and can reflect phylogenetic relationships, as different species coexisting in the same habitat often have species-specific microbiomes [26,27]. Studying the chigger microbiome is therefore crucial for understanding how biotic and abiotic factors across space, as well as the presence or absence of pathogenic microbes, shape their bacterial communities. By characterizing the microbiomes of different trombiculid mites and identifying their core and variable components, we may better understand efforts to control scrub typhus. Previous studies from Thailand [3,28,29], Saudi Arabia [30], Japan [31], and the United States [32] have identified over one hundred bacterial genera in free-living and host-associated chiggers. The bacterial composition varies with location and whether the chiggers are collected from hosts or free-living, highlighting the influence of both environment and host association. In Thailand, Takhampunya et al. [29] found that *Corynebacterium*, *Bacillus*, and *Staphylococcus* were dominant among host-associated chiggers and also detected pathogens such as *Bartonella*, *Borrelia*, and *Orientia*, as well as arthropod endosymbionts such as *Francisella*, *Coxiella*, *Wolbachia*, and *Candidatus* Cardinium. Chaisiri et al. [3] also reported a highly diverse microbiota, including *Orientia* and *Borrelia*, in chiggers collected from small mammals. In southwestern Saudi Arabia, Alkathiry et al. [30] reported Burkholderiaceae, *Corynebacterium*, *Candidatus* Cardinium, *Mycobacterium*, *Staphylococcus*, and *Wolbachia* as dominant taxa in chiggers from rodents. Similarly, Richardson et al. [32] found *Corynebacterium*, *Propionibacterium*, and *Methylobacterium* to be the most abundant taxa in chiggers from rodents in North Carolina, USA, along with potential pathogens *Orientia* and *Rickettsia*. In Japan,**Ogawa et al.** [31] identified obligate intracellular bacteria such as *Rickettsia*, *Wolbachia*, and *Rickettsiella* in free-living *Leptotrombidium scutellare* larvae.

Recently, we collected free-living chiggers in 10 different sites across the three ecological regions (i.e., Mountains, Piedmont, and Coastal Plain) of North Carolina, USA, and found that five sites (three in the Piedmont and two in the Coastal Plain) were infected with *Orientia* spp. [33]. However, that study focused exclusively on characterizing the occurrence of *Orientia* in US free-living chiggers. Another study examined the microbiome of rodent-associated chiggers from several counties in North Carolina and assessed how it differed across rodent and chigger species [32]. More recently, antibodies to *Orientia tsutsugamushi* were detected in serum samples from patients with eschars in NC [34]. In this study, we aimed to better understand the microbiome of free-living, unfed, chiggers collected from nine different geographic locations that encompass the three ecoregions of North Carolina. We hypothesize that the composition of the chigger microbiome may influence their vector competence for *Orientia* and other pathogenic bacteria. Furthermore, we hypothesize that the chigger microbiome is shaped by multiple factors, including geographic location, chigger species, host species, and infection status with *Orientia* spp. To address these hypotheses, we asked three questions: (1) Do chigger-associated bacterial communities differ in abundance and diversity among chigger species? (2) Does the presence of *Orientia* affect the abundance and diversity of these bacterial communities? and (3) Does the composition of the chigger microbiome vary by geographic location?

## Materials and methods

### Chigger collection

**Fig 1:**
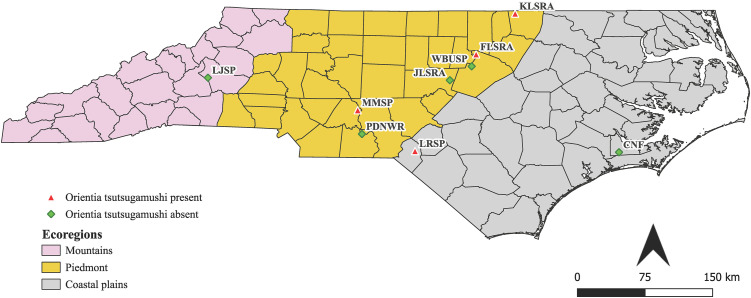
Map of sites in the different ecoregions of North Carolina, USA where free-living chiggers, with or without *Orientia*, were collected. Sites shown with a red triangle indicate where chiggers with *Orientia* were found. Chigger sites without *Orientia* are shown with a green diamond symbol.

In 2022, free-living chiggers were collected from nine sites in North Carolina, USA, spanning the states three ecoregions: the Coastal Plain, a flat, humid region in the east with sandy soils, wetlands, pine forests, low to moderate urbanization and large scale farming; the Piedmont, most urbanized among the three ecoregions, forms the central area of NC with rolling hills and clay-rich soils, mixed hardwood forests, and a moderate climate and agriculture; and the Mountain in the west, part of the Appalachian range, characterized by rugged terrain, cooler temperatures, and rich forests including spruce-fir at higher elevations. The Mountain ecoregion is relatively more rural and has limited, small scale agriculture. Aside from each ecoregion having distinct vegetation and wildlife, its environmental conditions also differ, shaping the local ecosystem. The sites by ecoregions were in the mountains, Lake James State Park, (LJSP); Piedmont, Morrow Mountain State Park (MMSP), Pee Dee National Wildlife Refuge (PDNWR), Jordan Lake State Recreation Area (JLSRA), William B. Umstead State Park (WBUSP), Falls Lake State Recreation Area (FLSRA); and Kerr Lake State Recreation Area (KLSRA), and Coastal Plain, Lumber River State Park (LRSP) and Croatan National Forest (CNF) (Fig 1). Maps were generated using QGIS (QGIS Development Team). The basemap shapefile used for spatial visualization was obtained from the DIVA-GIS database (https://diva-gis.org/data.html), which provides freely available geographic data for research purposes. Administrative boundary shapefiles from this source were used as the base layer onto which study data were plotted. No proprietary basemap services were used. Chigger collection was conducted under a wildlife collection license issued by the North Carolina Wildlife Resources Commission (permit #22-SC01252) and a scientific research and collecting permit issued by the North Carolina Division of Parks and Recreation (permit #2022_0642). The black tile method [35] was used to collect the free-living chiggers. Black tiles (10.8 cm each side) were placed on the ground, and after approximately one minute, we visually inspected them for chiggers. They appear as red moving objects (typically <  250 μm) on the tiles. When chiggers were observed, we carefully collected them with a 1.2 mm camelhair brush and transferred them into vials containing 95% ethanol for preservation. Chiggers from each location were stored separately as a group, and once identified, the DNA extraction was conducted separately on individual chiggers.

To ensure the inclusion of only the epidemiologically relevant larval (chigger) stage, all specimens were examined under a stereomicroscope. They were confirmed to be hexapod (six-legged) larvae, thereby distinguishing them from octopod (eight-legged) nymphal and adult stages. For species identification, representative specimens (10–15%) from each collection site were randomly selected, slide-mounted, and examined via compound microscopy (including oil immersion) using standard taxonomic keys [36–38]. Species identification was performed by Dr. Dac Crossley, Curator of the Acari collection at the Georgia Museum of Natural History.

### DNA extraction and chigger identification

Individual, free-living chiggers were surfaced sterilized [28] and total nucleic acids were extracted using a QIAGEN DNeasy Blood & Tissue Kit (QIAGEN, Valencia, CA, USA) according to the manufacturer’s instructions. A total of 120 chiggers were initially processed individually for DNA extraction. After surface sterilization, each chigger was placed into an individual sterile microcentrifuge tube containing approximately ten sterile 3-mm glass beads (Cat. No. 11-312A, Fisher Scientific) along with appropriate volumes of ATL buffer and Proteinase K, according to the DNA extraction kit protocol. Samples were homogenized using a FastPrep FP120 cell homogenizer (Thermo Electron Corporation, Waltham, MA, USA) and incubated at 56 °C for 1 h. Subsequently, AL buffer was added to each tube, mixed thoroughly, and incubated again at 56 °C for 1 h. DNA was then purified and eluted in 50 μL of nuclease-free water, and the eluate was stored at −20 °C until further use. DNA quantity and quality were initially assessed using NanoDrop spectrophotometry. Twenty-five samples did not yield detectable DNA and were excluded from further analysis. The remaining 95 samples were subjected to molecular identification using 18S ribosomal RNA gene primers and PCR [39]. The PCR products were Sanger sequenced at Eton Bioscience, Inc. (Research Triangle Park, NC, USA).

The sequences were queried using BLASTn against the NCBI nucleotide database to identify the chigger species using homologous sequences. Although 18S amplification was successful for all samples, 26 chigger samples produced ambiguous sequences, likely due to non-specific amplification. Consequently, 69 samples yielded high-quality sequences suitable for identification, while the 26 ambiguous samples were excluded from the final analysis. Sequence similarity thresholds of 99–100% were considered indicative of the same species. To reduce the risk of introducing foreign DNA, we used pre-sterilized tubes, pipettes, and filter tips that were UV-irradiated for 15 minutes prior to use. Dedicated pipettes and filter tips were maintained separately for DNA extraction and PCR setup to prevent cross-contamination. PCR reactions were prepared in a laminar flow hood.

### 16S rRNA gene amplification, Illumina library construction and sequencing

The 16S rRNA sequence library of individual chiggers was constructed following the Illumina 16S rRNA metagenomics sequencing library preparation protocol (Illumina, San Diego, CA, USA). The V3-V4 region of the bacterial 16S rRNA gene was then amplified using 341F/806R primers with previously used thermocycling conditions [40]. Each PCR assay included a negative control, which contained all reagents with DNA-free water as the template. Following amplification, all samples, including the negative control, were analyzed on a 1.5% agarose gel, and no amplification was detected in the negative control. Consequently, the negative controls were not processed for Illumina sequencing. The amplicons were purified using AMPure XP beads (AXYGEN, Big Flats, NY, USA). Then, index PCR was performed to barcode amplicons from each chigger. This was followed by another round of DNA purification using beads. Finally, samples were sent to the Microbiome Core Facility, University of North Carolina at Chapel Hill (Chapel Hill, NC, USA) for sequencing, on an Illumina MiSeq platform (300 bp paired-end reads).

### Bioinformatic and statistical analysis

Illumina FASTQ files were analyzed using the Quantitative Insights into Microbial Ecology 2 (QIIME2) software [41]. The Divisive Amplicon Denoising Algorithm 2 (DADA2) plugin was used for trimming and filtering, denoising, merging paired-end reads, and removing chimeras and amplicon sequence variant (ASV) inference [42]. Low-quality regions, forward and reverse reads were truncated at 280 and 220 nucleotides, respectively. Taxonomy assignments were performed using the Greengenes2 (2024.09) full-length backbone Naive Bayes classifier [43]. Alpha and beta diversity analyses were calculated using the QIIME 2 diversity core-metrics-phylogenetic plugin pipeline, and all the samples were rarefied to 25,000 sequences (based on the lowest read count among samples) to ensure an even sampling depth.

Alpha diversity (diversity within samples) was assessed using three metrics: the number of ASVs, which quantifies richness by counting unique ASVs; Shannon diversity, which accounts for both the abundance and evenness of ASVs [44]; and Faith’s phylogenetic diversity, which incorporates phylogenetic relatedness among ASVs [45]. The Kruskal–Wallis test (*p* ≤ 0.05) was performed to determine statistical significance of alpha-diversity metrics. Beta diversity (Bray–Curtis index) was calculated using the weighted UniFrac distance metric, which incorporates both phylogenetic relationships and relative abundances. EMPeror [46] was used for visualization of principal coordinate analysis (PCoA) plots. PERMANOVA tests (*p* ≤ 0.05) were used to test the statistical significance of β-diversity measurements. Statistical differences in bacterial community composition were also tested using PERMANOVA [47], which is particularly appropriate for analyzing unbalanced and non-orthogonal datasets. Analyses were conducted to compare: (i) the two most abundant chigger species (*E. splendens* and *Pseudoschoengastia* sp.), (ii) nine collection sites distributed across the ecoregions of NC (sample sizes for each site are provided in S1 Table), and (iii) *Eutrombicula splendens* chiggers infected versus uninfected with *Orientia.* We identified bacterial taxa that are differentially abundant between *Orientia*-infected and uninfected chiggers using ANCOM-BC (Analysis of Composition of Microbiomes with Bias Correction) in the q2-composition plugin, with compositional bias correction, unequal sequencing depths, and controls for false discovery rate to reduce false positives [48].

### Phylogenetic analyses of *Rickettsia, Listeria, Legionella, Staphylococcus* and *Streptococcus*

Phylogenetic trees were constructed to verify and/or refine the evolutionary relationships of *Rickettsia*, *Listeria*, *Legionella*, *Staphylococcus*, and *Streptococcus* sequence variants identified in this study, using closely related sequences retrieved from the NCBI database through BLASTn analysis (accessed on June 12, 2025). Maximum-likelihood (ML) phylogenetic trees were generated using the Kimura two-parameter model in the MEGA 11 software [49]. Bootstrap support values were calculated based on 1,000 resampling iterations. The phylogenetic analysis of *Orientia* has already been described by Chen et al. [33].

## Results

### Chigger identification

A total of 69 chigger samples (individual chiggers) used in this study were collected from nine recreational parks in North Carolina, USA (Fig 1). Six to nine chiggers were used from each collection site for this study. To identify the chiggers, 18S ribosomal RNA gene amplicon sequences from each were analyzed by BLASTn. Fifty-four samples showed 99–100% identity with homologous sequences of *E. splendens* (accession no. KY922159); four samples showed 100% identity with homologous sequences of *E. tinami* (accession no. ON870584); and eleven samples showed 95.2–96.3% identity with homologous sequences of *Pseudoschoengastia petrolinensis*. In the latter case because the level of similarity is below the species-level threshold for 18S rRNA (which requires nearly identical sequences due to the conserved nature of the marker), species-level assignment was not reliable. However, morphological characteristics were consistent with the genus *Pseudoschoengastia*. Therefore, these specimens were assigned only to the genus level.

Summary of chigger sampling and identification is in S1 Table.

### Overall sequencing results

**Fig 2:**
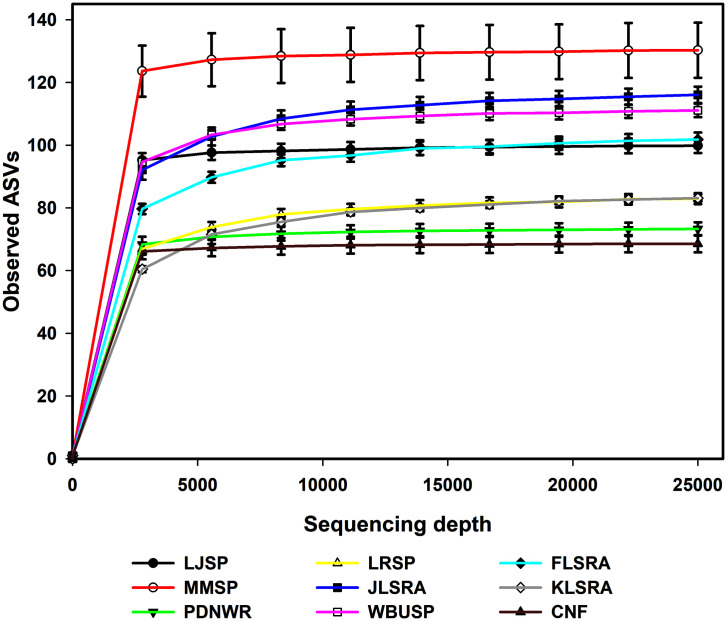
Rarefaction curves of the average number of observed features of free-living chiggers, *Eutrombicula splendens*, among nine different collection sites. Error bars represent the standard error of the means. Each symbol corresponds to chiggers collected from a specific site. LJSP, Lake James State Park; MMSP, Morrow Mountain State Park; PDNWR, Pee Dee National Wildlife Refuge; LRSP, Lumber River State Park; JLSRA, Jordan Lake State Recreational Area; WBUSP, William B. Umstead State Park; FLSRA, Falls Lake State Recreational Area; KLSRA, Kerr Lake State Recreational Area; CNF, Croatan National Forest.

**Fig 3:**
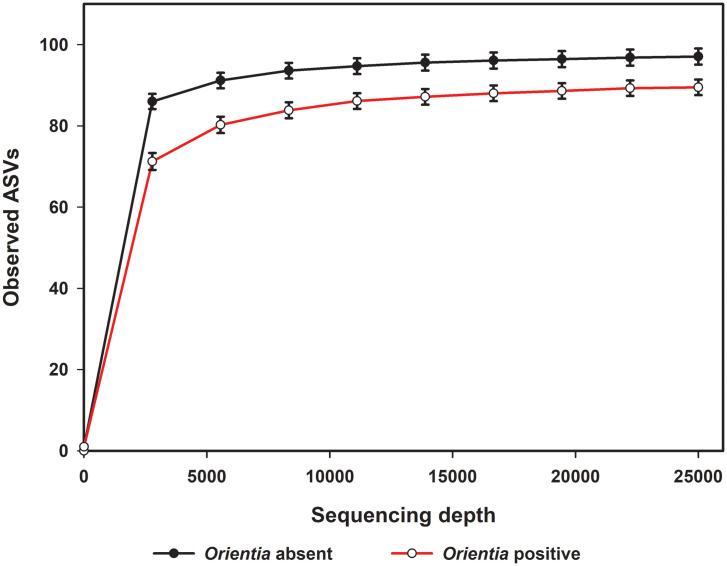
Rarefaction curves of the average number of observed features of free-living *Eutrombicula splendens* chiggers with or without *Orientia.* Error bars represent the standard error of the means.

A total of 11,548,050 raw sequencing reads were obtained across the 69 chigger samples using Illumina 16S rRNA sequencing. Following quality filtering and denoising with the DADA2 algorithm, 6,554,232 high-quality reads were retained, with a median of 108,534 reads per sample. A total of 3,575 ASVs were obtained and used in downstream analyses. The median observed ASVs per sample was 91. The percentages of ASVs classified were phylum (100%), class (100%), order (99.2%), family (98.2%), and genus (93.0%). These sequencing reads were taxonomically classified into 38 bacterial phyla, 77 classes, 213 orders, 407 families, and 797 genera, reflecting a diverse and complex bacterial community associated with the chigger samples. The rarefaction curves of observed ASVs reached plateaus for all samples, indicating that a sequencing depth of 25,000 was adequate to retrieve most of the bacterial taxa in our samples (Fig 2, Fig 3, S1 Fig).

### Alpha diversity

**Comparison among chigger species:** Three metrics were used to measure the bacterial species diversity constituting the chigger microbiome, namely observed ASVs, the Shannon’s diversity index and the Faith’s phylogenetic diversity index. Due to the limited number of samples available for *E. tinami* (n = 4), these were excluded from the diversity analysis. No significant differences in Observed ASVs, which focuses on the richness in terms of the number of unique ASVs, were detected between *E. splendens* and *Pseudoschoengastia* sp. (Kruskal–Wallis test, H = 1.68, *p* = 0.19; S2A Fig). Similarly, Shannon diversity, which accounts for species richness and evenness, revealed no significant differences between the two groups (Kruskal–Wallis test, H = 0.13, *p* = 0.71; S2B Fig). The Faith’s phylogenetic diversity, which incorporates phylogenetic relatedness, also did not differ significantly between species (Kruskal–Wallis test, H = 0.13, *p* = 0.71; S2C Fig).

**Fig 4:**
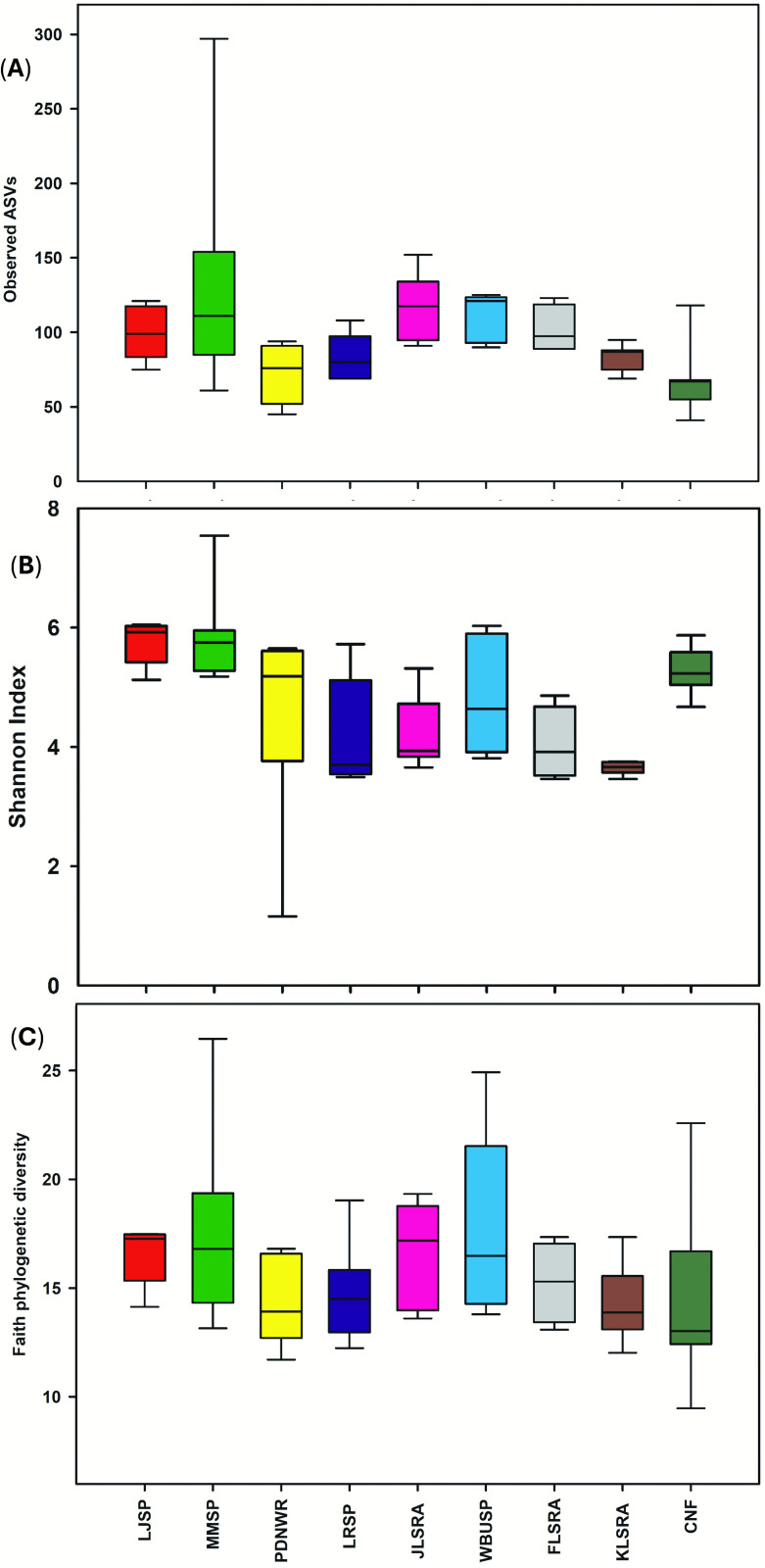
Alpha diversity measures of the bacterial microbiomes of free-living chiggers *Eutrombicula splendens* among nine different collection sites. (A) Observed ASVs, (B) Shannon diversity and (C) Faith’s phylogenetic diversity. LJSP, Lake James State Park; MMSP, Morrow Mountain State Park; PDNWR, Pee Dee National Wildlife Refuge; LRSP, Lumber River State Park; JLSRA, Jordan Lake State Recreational Area; WBUSP, William B. Umstead State Park; FLSRA, Falls Lake State Recreational Area; KLSRA, Kerr Lake State Recreational Area; CNF, Croatan National Forest.

**Effect of geographic location:** We selected only *E. splendens* samples, the most abundant and present in all nine sites among the three chigger species, to test whether microbiome composition varied across sites. The number of observed ASVs differed significantly among the sites (Kruskal–Wallis test, H = 25.33, *p* = 0.001; Fig 4A). Similarly, the Shannon diversity index indicated significant differences in diversity across sites (Kruskal–Wallis test, H = 30.16, *p* = 0.002; Fig 4B). In contrast, the Faith’s phylogenetic diversity metric did not reveal significant differences among locations (Kruskal–Wallis test, H = 12.28, *p* = 0.14; Fig 4C).

**Fig 5:**
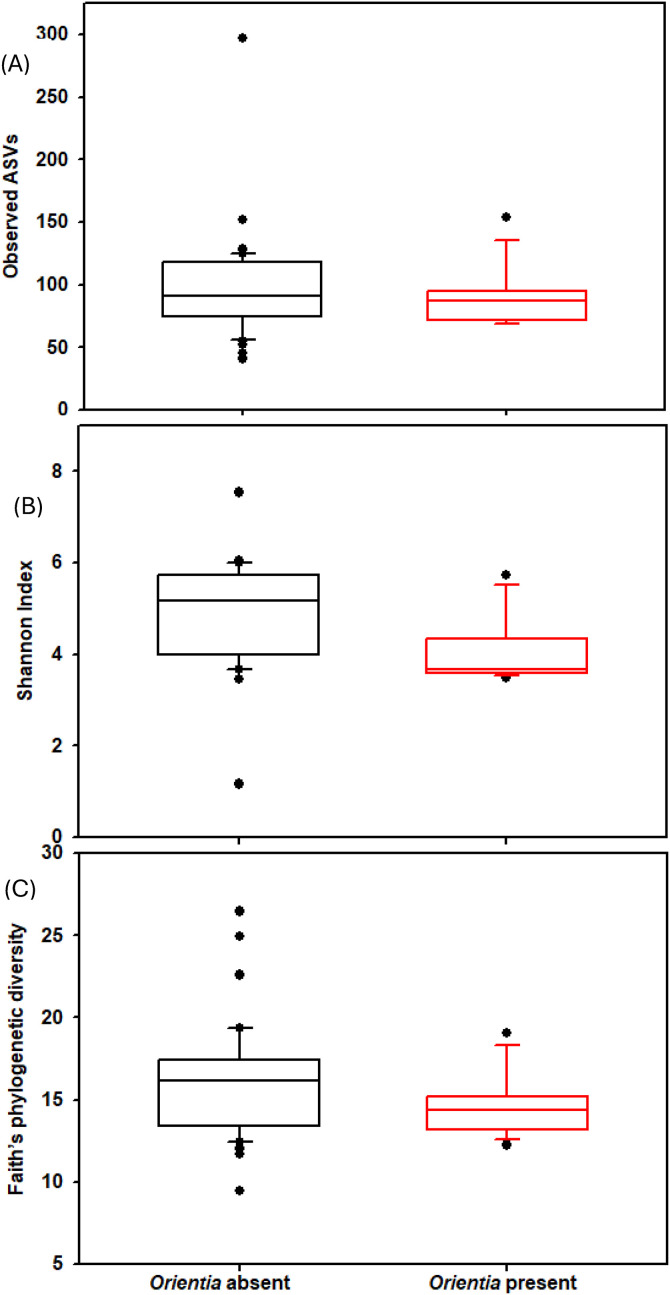
Alpha diversity measures of the microbiomes of free-living chiggers, *Eutrombicula splendens*, with or without *Orientia.* (A) Observed ASVs, (B) Shannon diversity and (C) Faith’s phylogenetic diversity.

We also investigated whether the presence of *Orientia* was associated with differences in microbiome diversity within *E. splendens*. No significant differences were found between *Orientia*-positive and -negative samples based on Observed ASVs (Kruskal–Wallis test, H = 0.69, *p* = 0.41; Fig 5A) or the Faith’s phylogenetic diversity (Kruskal–Wallis test, H = 1.13, *p* = 0.29; Fig 5C). However, a significant difference was observed using the Shannon diversity index (Kruskal–Wallis test, H = 10.54, *p* = 0.001; Fig 5B).

### Beta diversity

**Fig 6:**
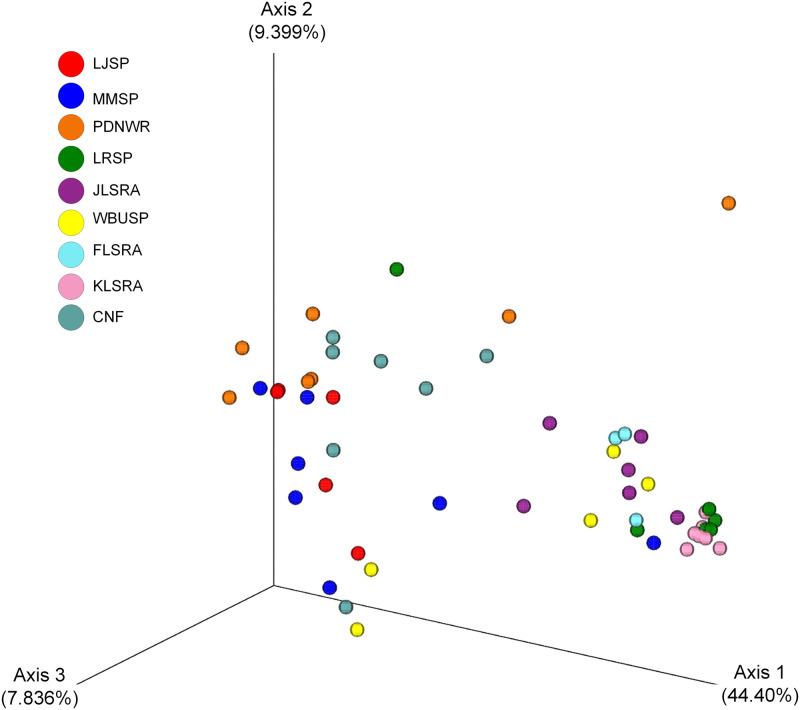
Principal Coordinate Analysis (PCoA) of the bacterial composition of free-living chiggers, *Eutrombicula splendens*, among the nine different collection sites. Each site is represented by a different color. Analysis was based on the weighted Unifrac metric**.** LJSP, Lake James State Park; MMSP, Morrow Mountain State Park; PDNWR, Pee Dee National Wildlife Refuge; LRSP, Lumber River State Park; JLSRA, Jordan Lake State Recreational Area; WBUSP, William B. Umstead State Park; FLSRA, Falls Lake State Recreational Area; KLSRA, Kerr Lake State Recreational Area; CNF, Croatan National Forest.

**Fig 7:**
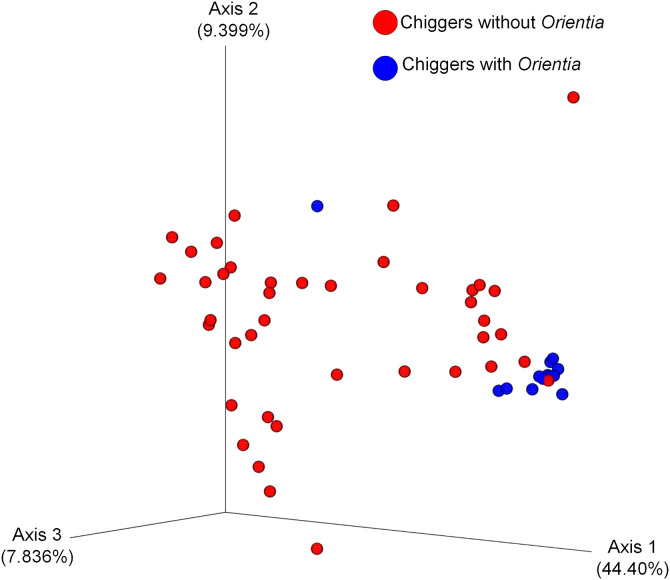
Principal coordinate analysis (PCoA) of the bacterial microbiome of free-living chiggers, *Eutrombicula splendens*, infected and uninfected with *Orientia.*

Beta diversity was assessed using weighted UniFrac distances. By species, Principal Coordinates Analysis (PCoA) revealed no distinct clustering and no significant differences between *E. splendens* and *Pseudoschoengastia* sp. (S3 Fig; PERMANOVA, *p* = 0.125). In contrast, significant differences in bacterial microbiome composition were observed among *E. splendens* samples from the nine sites (Fig 6; PERMANOVA, *p* = 0.001), as well as between samples positive and negative for *Orientia* (Fig 7; PERMANOVA, *p* = 0.001). Pairwise PERMANOVA comparisons among the nine collection sites for *E. splendens* are summarized in S2 Table. Again, beta diversity for *E. tinami* was not analyzed due to its small sample size.

### Bacterial community composition

**Fig 8:**
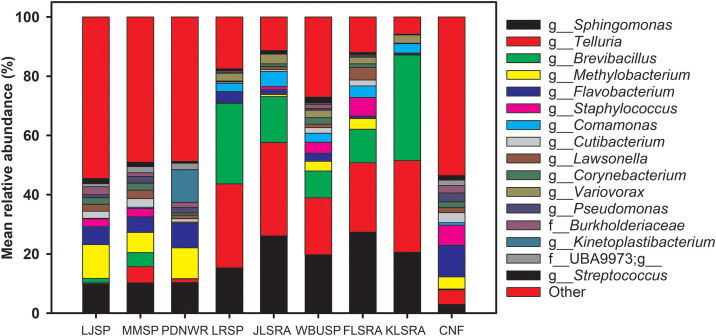
Relative abundance of major bacteria at genus level for *Eutrombicula splendens* chiggers collected from nine different sites in North Carolina, NC. Some of the taxa were only identified to class or family level. In the legend, “Other” refers to the taxa with relative abundance lower than 1%, “f_” represents family and “g_” represents genus. LJSP, Lake James State Park; MMSP, Morrow Mountain State Park; PDNWR, Pee Dee National Wildlife Refuge; LRSP, Lumber River State Park; JLSRA, Jordan Lake State Recreational Area; WBUSP, William B. Umstead State Park; FLSRA, Falls Lake State Recreational Area; KLSRA, Kerr Lake State Recreational Area; CNF, Croatan National Forest.

The four most abundant bacterial genera across all chigger samples from *E. splendens* were *Sphingomonas* (15.53%), *Telluria* (13.78%), *Brevibacillus* (10.59%), and *Methylobacterium* (5.43%). Fig 8 displays the relative abundance of genus level bacterial taxa in chiggers collected from the nine sites. *Brevibacillus*, *Telluria*, and *Sphingomonas* were particularly abundant in samples from LRSP (27.2%, 28.4%, and 15.3%, respectively), JLSRA (15.5%, 31.5%, and 26.1%), WBUSP (9.1%, 19.3%, and 19.7%), FLSRA (11.3%, 23.4%, and 27.4%), and KLSRA (35.6%, 31.0%, and 20.5%). In contrast, *Flavobacterium* showed higher relative abundance in samples from LJSP (6.1%), MMSP (5.3%), PDNWR (8.3%), and CNF (10.7%).

Additionally, we detected a potentially pathogenic genus *Rickettsia*, found in 4.3% of samples, with a median relative abundance of 0.15% in the infected samples. Genus *Listeria* was detected in 2.8% of samples, with a median relative abundance of 0.18% among the infected samples. Genus *Legionella* was detected in 13.0% of samples, with a median relative abundance of 0.05% among the infected samples. Genus *Staphylococcus* was detected in 87.0% of samples, with a median relative abundance of 1.6% among the infected samples. Genus *Streptococcus* was detected in 75.4% of samples, with a median relative abundance of 0.65% among the infected samples.

**Fig 9:**
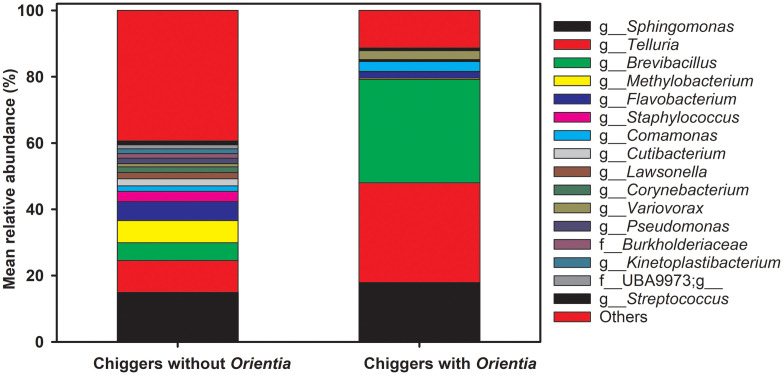
Relative abundance of major bacteria at the genus level for free-living *Eutrombicula splendens* chiggers with or without *Orientia.* Some of the taxa were only identified to class or family level. In the legend, “Other” refers to the taxa with relative abundance lower than 1%, “f_” represents family and “g_” represents genus.

The relative abundance of bacterial taxa in chiggers with and without *Orientia* are shown in Fig 9. *Brevibacillus*, *Telluria*, and *Sphingomonas* were the dominant genera in *Orientia*-positive chiggers (31.2%, 30.1%, and 17.9%, respectively) and were also present, though at lower abundances, in *Orientia*-negative chiggers (5.3%, 9.6%, and 14.9%, respectively). The bacterial community in chiggers without *Orientia* appeared more diverse, with no single genus overwhelmingly dominating. Differentially abundant taxa between *Orientia*-positive and *Orientia*-negative chiggers were identified using the ANCOM-BC test, with results presented in S4 Fig. In *Orientia*-positive chiggers, the genera *Brevibacillus*, *Variovorax*, *Delftia*, *Paenibacillus*, *Cohnella*, *Telluria* and *Sphingomonas*, and the family Paenibacillaceae were significantly more abundant. In contrast, *Propionibacterium* was the most significantly depleted genus compared to *Orientia*-negative chiggers (Fig 9).

### Phylogenetic analyses

To further identify *Rickettsia* spp. sequences, one ASV classified within the genus *Rickettsia* and 14 closely related species identified via BLASTn searches were used to construct phylogenetic trees using the maximum-likelihood (ML) method (S5 Fig). The phylogenetic analysis indicated that the *Rickettsia* ASV clustered with the *R. bellii* strain 369L42-1 (NR_036774) and the *R. akari* strain MK Kaplan (NR_029154) with 100% sequence similarity, and with *R. australis* (L36101) with 99.75% similarity. In genus *Listeria*, three ASVs were classified, and 17 closely related species were included in a phylogenetic tree constructed using the same ML approach (S6 Fig). The analysis revealed that *Listeria* ASV #1 clustered with *Listeria monocytogenes* (MK014484) with 100% similarity. *Listeria* ASVs #2 and #3 were most closely related to the *L. immobilis* strain FSL L7-1519 (NR_181103), showing over 98.13% sequence similarity. In the genus *Legionella*, 8 ASVs were identified. Phylogenetic analysis (S7 Fig) revealed that 6 AVS were closely related to *Legionella pneumophila* and *L. fallonii* (97.34%–97.98%). Two ASVs were closely related to *L. massiliensis* (97.66%–97.89%). In *Staphylococcus*, 48 ASVs were identified, with pairwise sequence similarities ranging from 97.6% to 99.9%. Phylogenetic analysis (S8 Fig) revealed three major groups: Group 1 (25 ASVs) was closely related to *S. epidermidis* and *S. capitis* (98.36%–99.9%); Group 2 (16 ASVs) to *S. aureus S. warneri* and *S. ratti* (98.23%–99.9%); and Group 3 (7 ASVs) to *S. pettenkoferi* (98.1%–100%) showing sequence similarity with their closest relatives. In *Streptococcus*, 38 ASVs were identified, with pairwise similarities ranging from 96.4% to 100%. Phylogenetic analysis (S9 Fig) grouped them into six clusters: Group 1 (7 ASVs) was closely related to *S. vulneris* (98.5%–100%), Group 2 (8 ASVs) to *S. australis* (99.4%–99.9%), Group 3 (3 ASVs) to *S. sanguinis* (99.53%–100%), Group 4 (7 ASVs) to *S. lingualis* (97.1%–99.8%), Group 5 (6 ASVs) to *S. anginosus* (90.06%–99.9%) and Group 6 (8 ASVs) to *S. parasalivarius* (98.6%–99.8%).

## Discussion

Chiggers are widespread on the planet and found in diverse ecological regions [3,50,51]. However, little to no knowledge exists on the microbiome of chiggers in the United States. The goal of our work here was to determine whether chigger species, ecological region and *Orientia* infection affect the microbiome of free-living chiggers in the US in North Carolina. Understanding more about the microbiome of free-living chiggers and the factors that influence it will improve our knowledge of the risks these mites pose to humans in North America.

*Orientia* spp. which cause the human disease, scrub typhus, has traditionally been associated with transmission through the bites of free-living chiggers in the Asia-Pacific region. However, more recent studies have detected *Orientia* in the Middle East and South America [17,50,52,53], raising the possibility of its presence in North America as well. We recently reported, for the first time in North America, specifically in North Carolina, USA, the presence of *Orientia* in both free-living chiggers [33] and rodent attached chiggers [32]. This challenges the current paradigm in the United States, which holds that chiggers do not transmit disease-causing microbes to humans. Our findings suggest that *Orientia* is likely circulating enzootically within the U.S., warranting further investigation into its potential public health impact. In addition to *Orientia*, chiggers have been found to harbor other pathogens, including *Rickettsia*, *Borrelia*, *Bartonella*, and *Anaplasma* spp., as well as hantavirus [50], though their role as vectors of these microbes to humans remains uncertain.

### Distribution of different free-living chigger species in NC

In this current study, we haphazardly surveyed for free-living chiggers on a west to east transit covering three ecological areas, i.e., the mountains, the Piedmont and the coastal plain of NC (Fig 1). All collections were in public parks where people are exposed to chigger bites. *Eutrombicula splendens* was found in all three ecoregions and at all collection sites except for the Pee Dee National Wildlife Refuge (PDNWR; Fig 1) in the Piedmont ecoregion suggesting that *E. splendens* is a common chigger species not restricted to a specific ecoregion of NC. The *E. splendens* within each ecoregion are more local than ephemeral because the distance from our western mountain collection sites to close to the ocean in the east is 518–766 km with very different types of vegetation and climates and can therefore not be transported from the mountain ecoregion in the west to the eastern coastal ecoregion. *Pseudoschoengastia* sp*.*, which was first recorded in Brazil in 2019 [54] but never in the USA, was not found in our mountain site (S1 Table) but was collected North to South in the piedmont as well as the Coastal regions. In contrast, *E. tinami* was found only at our mountain site and at two of our Piedmont sites. Alternatively, the low number collected and at limited sites could indicate that the species are rare or were simply missed based on our haphazard sampling design. The season we collected these chiggers probably affected our detection of these trombiculid mite species. Some chigger species prefer one season over others [55]. This may also be a reason for observing low abundances of *Pseudoschoengastia* sp. and *E. tinami* compared to *E. splendens*. A more thorough surveillance design, which will incorporate the effect of seasonal variations and site locations (i.e., north, mid, or south), could probably provide a better understanding of the mite species distribution across the state. Chaisiri et al. [3] in Thailand found that chigger species richness was positively correlated with latitude and negatively correlated with human land use. Chigger diversity was also higher in the dry season. Another study in China [56] found a humped shape pattern between chigger diversity on hosts and latitude. The negative effect of human land use on chigger species richness was attributed to loss of biodiversity with increasing anthropogenic activities such as agriculture and urbanization [3].

### Differences in the microbiomes between chigger species

*Orientia*-infection was found in 13 *E. splendens* and one *Pseudoschoengastia* sp. collected from three sites in the Piedmont and one site in the coastal plain (Fig 1). All the four *E. tinami* were negative for *Orientia*, but this may be due to their small sample size (n = 4) and hence, their exclusion from the microbiome comparisons. No differences in the microbiomes were found between *E. splendens* and *Pseudoschoengastia* sp. based on all three alpha diversity measures, i.e., observed ASV numbers (S2-A Fig), Shannon diversity (S2-B Fig), and the Faith’s phylogenetic diversity (S2-C Fig). Similar to alpha diversity metrics, the beta diversity analysis also showed no significant difference between *E. splendens* and *Pseudoschoengastia* sp*.* (S3 Fig). This result was unexpected, as the comparison involved different chigger genera. However, they were collected at the same geographical sites suggesting their environmental exposure to bacteria would be the same, and this might explain their similar microbiome. These results also suggest they have similar mechanisms to internalize bacteria from their common habitat. There is clear evidence of species-specific microbial signatures in some ticks [57], while others have remarkable similarities across different species and genera (where ticks share similar habitats, host ranges, or physiological characteristics). Namina et al. [58] found significant overlap in the core microbiomes of *Ixodes ricinus* and *I. persulcatus* which was attributed to their common environmental exposure and feeding behavior. Thus, the similar diversity metrics observed in *E. splendens* and *Pseudoschoengastia* sp*.* may reflect a stable, functionally constrained microbiome derived from the environment rather than taxon-specific microbial diversity.

### Geographical variations in the *E. splendens* microbiome

Most of the chiggers we collected were *E. splendens* and occurred in all three ecoregions. We therefore used this species to examine whether there were geographical differences in the free-living chigger microbiomes. The results were mixed, i.e., the numbers of observed ASVs were different (Fig 4A) across ecoregions and so was the Shannon diversity index (Fig 4B), but there was no difference in the Faith’s phylogenetic diversity metric (Fig 4C). These findings were not too surprising since we do not expect phylogenetic variations within the same species. There exist differences in bacterial microbiome alpha diversity across different spaces. Previous studies have shown before that latitudinal gradients were positively correlated with increased chigger species richness [3,59]. However, research on the impact of the environment on the microbiome of free-living chiggers is scarce and most of what we do understand is from host associated chiggers. This is mainly because the convenience of collecting attached chiggers from trapped rodents is greater than actively searching for highly aggregated, free-living chiggers that walk across small black tiles. For host associated chiggers collected from small mammals in oil palm in Malaysia (which included chiggers from the genus *Eutrombicula* and *Leptotrombidium*), the dry season increased chigger populations and *Orientia* infection, thereby increasing the risk for scrub typhus [60]. Possible explanations are a change in the chigger microbiome and/or in chigger host interactions across seasons. Elliott et al. [61] found that *O*. *tsutsugamushi-*positive hosts and chiggers were related to the ending dry and wet seasons. It also was shown that human scrub typhus infection rates were associated with higher relative humidity [62] although the mechanism is unknown. Our group has recently found geographical differences in the microbiome of rodent collected chiggers within the same ecoregions of NC [32]. This study showed that the highest bacterial species richness and percentage of unique ASVs were from the hispid cotton rat in the eastern part of the Piedmont ecoregion. However, other parts of the Piedmont had low but unique ASVs. Chiggers collected in the mountain ecoregion close to the western Piedmont had similar alpha diversity to those that were some distance away in the Piedmont. Environment can affect host microbiomes and in turn that of host-associated chiggers [32,63]. In addition, specific chigger endosymbionts [28,64], the digestive system microbiome, and co-feeding on the same animal can also affect the internal bacterial community. For example, relative to the latter, *O. tsutsugamushi* was horizontally transferred from infected chiggers to uninfected chiggers of a different species while both were feeding on the same host [65,66]. Furthermore, *Orientia* infection may also be a factor influencing the chigger internal microbiome (discussed later), and Richardson et al. [32] found that chiggers collected from hispid cotton rats had greater bacterial diversity compared to chiggers collected from the white-footed mouse. Our research here makes a significant advancement in understanding the impact of habitat on the microbiome of free-living chiggers, and clearly more work is needed to understand the factors that shape this larval mite microbiome. In NC, the only positive chiggers for *Orientia* were found close to the border between the Piedmont and coastal plain. This coastal plain and the Piedmont area is comprised of flat land and used for agriculture, implying that human land use changes may be positively driving *Orientia* infection rates among chiggers in NC.

### Bacterial composition of *Orientia* infected and non *Orientia* infected chiggers

The microbiome of *Orientia* infected chiggers was clearly different from chiggers not infected with *Orientia* (Fig 9). *Brevibacillus*, *Telluria* and *Sphignomonas* were abundant in *Orientia* positive chiggers at 31.2%, 30.1%, and 17.9%, respectively, as compared to *Orientia* negative chiggers at 5.3%, 9.6%, and 14.9%, respectively. The bacterial community in the latter was more diverse with no single genus overwhelmingly dominating the community. The ANCOM-BC test showed *Brevibacillus*, *Variovorax*, *Delftia*, *Paenibacillus*, *Cohnella*, *Telluria*, Paenibacillaceae, and *Sphingomonas* were enhanced when *Orientia* was present, while *Propionibacterium* was less abundant when compared to chiggers without *Orientia*. These observations are correlative and do not prove a biological function. This impact of *Orientia* presence on the microbiome was shown before in chiggers. Infection with *O. tsutsugamushi* influenced the abundance and diversity of other bacterial taxa in *L. imphalum*. A mutualistic relationship was observed between *O. tsutsugamushi* and a novel Amoebophilaceae species in a laboratory colony of chiggers in Thailand [28]. This discovery in free living chiggers in NC and its similarity to a similar finding for chiggers in Thailand, strongly suggest *Orientia* infection might require co-infection with other bacteria, likely due to both contributing to each other, factors that sustain their chigger infection. This co-dependence may also be important at some level in vertebrate host infections. This clearly needs further study.

The free-living chigger microbiome is expected to be different from that of host-associated chiggers due to the possible acquisition of internal and external bacteria from the host in variable proportions. A microbiome analysis of rodent collected chiggers in Saudi Arabia found the dominant genera to be *Corynebacterium*, *Mycobacterium*, *Staphylococcus*, *Candidatus* Cardinium, *Burkholderiaceae* and *Wolbachia* along with potential pathogenic *O*. *chuto* and a *Coxiella burnetii*-like microbe in the chiggers, *Ericotrombidium kazeruni* and *Pentidionis agamae* [30]. Chaisiri et al. [3] found as dominant in rodent collected Thailand chiggers *Sphingobium*, *Mycobacterium*, Neisseriaceae and Bacillales. They also found *O*. *tsutsugamushi*, *Borrelia* spp. (in *L. delicense*), *Staphylococcus* and *Haemophilus parainfluenzae* from multiple chigger species. Richardson et al. [32] studying the microbiome of chiggers collected from hispid cotton rats, *Sigmodon hispidus* and white-footed mouse, *Peromyscus leucopus*, in North Carolina, USA, found that the chigger microbiomes were atypical, except for those from Saudi Arabia [30] which were the most similar to NC. This similarity could reflect similarity in the microbial environment of the rodent hosts between the two geographically distinct collection sites Only a few studies have looked at the effect of factors, such as, life stage and the presence or absence of pathogens [28], host [3,32], seasonality, habitat and the environment [3], on the chigger microbiome.

### Presence of other potentially pathogenic bacteria

We detected *Rickettsia* spp. in only three chiggers (4.3%) across all *E. splendens* collections from nine sampling locations in NC. In contrast, Richardson et al. [32] reported a higher prevalence (16.7%) of *Rickettsia* spp. in host-associated chiggers. Field-collected chiggers have also been found infected with *Rickettsia* spp. in Thailand [67], Taiwan [20], and NC, US [23]. In the US study, the *Rickettsia*-positive chiggers were collected, and sequence analyses revealed high similarity to *Rickettsia bellii*, *R. australis*, and *R. akari* [23]. These species are known human pathogens typically transmitted by ticks, mites, or fleas. These findings raise further questions about the role of chiggers in the transmission of *Rickettsia* spp. to humans, particularly regarding pathogens more commonly linked to other arthropod vectors.

We also detected *Legionella* spp. in 9 chiggers collected during our survey. This was unexpected, raising questions about the potential role of chiggers in the ecology of these bacteria. While *Legionella* is predominantly known as an aquatic bacterium residing within protozoa [68], its presence has been sporadically reported in association with other terrestrial and arthropod systems, underscoring its adaptability. More critically, *Legionella* is known to survive and replicate within free-living amoebae, which themselves can be found in association with various arthropods (e.g., in tick guts or mosquito larvae) as part of their environmental microbiome [69]. This indirect association suggests that arthropods could potentially carry *Legionella*-laden amoebae or *Legionella* cells acquired from environmental sources. To our knowledge, this study may represent the first report of *Legionella pneumophila* detection in chiggers, highlighting the potential need to expand surveillance and preventive efforts beyond established rodent-borne pathogens to include *L. pneumophila* as a possible zoonotic threat.

A noteworthy observation was the substantial amount of genus *Streptococcus* and *Staphylococcus* present in free living NC chiggers. *Staphylococcus*, a potentially pathogenic genus that was the most prevalent taxa among all samples with a higher frequency in both *Orientia-*infected and uninfected chiggers. The association of the *Staphylococcus* genus with chiggers was significant and aligns with a growing body of literature demonstrating the widespread association of these bacterial groups with various arthropods. Numerous studies have identified *Staphylococcus* species in various tick species, often within their gut, salivary glands, and even transovarial movement [70,71]. Tick bites, in general, are recognized as potential entry points for secondary bacterial infections, including those caused by *Staphylococcus aureus* [72]. This aligns with previous studies that found *Staphylococcus* in *Leptotrombidium imphalum* and *Amblyomma cajennense* [28,73,74]. While the role of *Staphylococcus* in the chigger microbiome remains unclear, it may be linked to the horizontal transmission of chigger-borne pathogens from the host skin microbiome [75]. Similarly, *Streptococcus* spp. have also been detected in ticks, although they are not typically considered primary tick-borne disease agents in the same way as *Borrelia* or *Rickettsia*. Microbiome studies of various tick species, including *Ixodes scapularis*, reveal diverse bacterial communities, which can include *Streptococcus* as part of the acquired environmental or host-derived flora [76,77]. Similar to tick bites, chigger bites that break the skin can become secondarily infected by common skin bacteria, including *Streptococcus* spp., leading to localized cellulitis or have other infections [72]. While direct research on *Streptococcus* persistence or replication within tick tissues is less extensive compared to other pathogens, their presence indicates exposure and potential for transient carriage.

## Conclusions

We found multiple species of larval trombiculid mites chiggers in NC with *E. splendens* and *Pseudoschoengastia* sp. dominant across ecoregions. *Orientia*-infected chiggers in NC were exclusive to the border between the Piedmont and coastal plain. It is not clear whether this is a consequence of our limited, haphazard sampling or the true effect of habitat, the environment, host effects, and/or a founder effect. The chigger microbiome was similar by multiple measures between the two widely distributed chigger species from different genera. In the dominant chigger species, *E. splendens*, the microbiome changed by most measures based on NC geography, and the microbiome diversity in these chiggers was greatly reduced by *Orientia* infection with clear dominant non-*Orientia* bacteria. This association of *Orientia* with a particular subset of bacterial species suggests that the establishment of *Orientia* in chiggers might be affected by the bacterial community composition, which in turn might also be affected by the local environmental conditions. *Orientia* vertebrate host infection in North America including NC has not been examined yet. Since the current public recommendation in the US is that chiggers do not transmit microbes that cause human disease and yet chiggers are known to cause human disease in Asia, the Middle East and South America and free-living and rodent-attached chiggers in the US are infected with *Orientia*, more research is needed to better understand the chigger microbiomes in the US and its impact on vertebrates.

## Supporting information

S1 Table Summary of chigger sampling and identification results. The nine collection sites are arranged from west to east of North Carolina, USA. Chigger identification was conducted using 18S ribosomal RNA gene amplicon sequences. The number of chiggers and the percent identity are included in parentheses after each species.
(DOCX)

S2 Table Pairwise PERMANOVA analysis results for free-living chiggers, *Eutrombicula splendens*, collected from nine different locations based on the weighted UniFrac. LJSP, Lake James State Park; MMSP, Morrow Mountain State Park; PDNWR, Pee Dee National Wildlife Refuge; LRSP, Lumber River State Park; JLSRA, Jordan Lake State Recreational Area; WBUSP, William B. Umstead State Park; FLSRA, Falls Lake State Recreational Area; KLSRA, Kerr Lake State Recreational Area; CNF, Croatan National Forest.
(DOCX)

S1 Fig Rarefaction curves of the mean number of observed ASVs in *Eutrombicula splendens* and *Pseudoschoengastia* sp. chiggers. Error bars represent the standard error of the mean.
(TIF)

S2 Fig Alpha diversity measures the microbiome in *Eutrombicula splendens* and *Pseudoschoengastia* sp*.* chiggers. (A) Observed ASVs, Shannon diversity, (B) Shannon diversity, and (C) Faiths phylogenetic diversity.
(TIF)

S3 Fig Principal coordinate analysis (PCoA) of the bacterial composition of *Eutrombicula splendens* and *Pseudoschoengastia* sp. chiggers. Analysis was based on the weighted Unifrac metric.
(TIF)

S4 Fig Plot of log fold change in taxon abundance for taxa which significantly differed (p < 0.001) between free-living chiggers *Eutrombicula splendens* with or without *Orientia.* (TIF)

S5 Fig Molecular phylogenetic analysis of *Rickettsia* amplicon sequence variants (ASVs) identified in this study (in red text) and closest related species. Phylogenetic tree constructed by the Maximum-likelihood (ML) method based on the 16S rRNA gene. The *Orientia tsutsugamushi* strain (NR 025860) was included as an outgroup.
(TIF)

S6 Fig Molecular phylogenetic analysis of *Listeria* amplicon sequence variants (ASVs) identified in this study (in red text) and closest related species. Phylogenetic tree constructed by the Maximum-likelihood (ML) method based on 16S rRNA gene. The *Brochothrix campestris* strain (NR044824) was included as an outgroup.
(TIF)

S7 Fig Molecular phylogenetic analysis of *Legionella* amplicon sequence variants (ASVs) identified in this study (in red text) and closest related species. Phylogenetic tree constructed by the Maximum-likelihood (ML) method based on 16S rRNA gene.
(TIF)

S8 Fig Molecular phylogenetic analysis of *Staphylococcus* amplicon sequence variants (ASVs) identified in this study (in red text) and closest related species. This tree is collapsed with nodes organized by sequence differences of 0.002. Phylogenetic tree constructed by the Maximum-likelihood (ML) method based on 16S rRNA gene.
(TIF)

S9 Fig Molecular phylogenetic analysis of *Streptococcus* amplicon sequence variants (ASVs) identified in this study (in red text) and closest related species. This tree is collapsed with nodes organized by sequence differences of 0.002. Phylogenetic tree constructed by the Maximum-likelihood (ML) method based on 16S rRNA gene.
(TIF)
