# Supplementary material for: Geographic and Orientia infection status influence on the bacterial microbiome of free-living chiggers in North Carolina, USA
Source: PLoS One. 2026 Jul 8;21(7):e0353174. doi: 10.1371/journal.pone.0353174 (PMC13345271; doi:10.1371/journal.pone.0353174)
Supplement: S1 Table — The nine collection sites are arranged from west to east of North Carolina, USA. Chigger identification was conducted using 18S ribosomal RNA gene amplicon sequences. The number of chiggers and the percent identity are included in parentheses after each species. (DOCX) [file pone.0353174.s001.docx]

**Table S1.** Summary of chigger sampling and identification results. The nine collection sites are arranged from west to east of North Carolina, USA. Chigger identification was conducted using 18S ribosomal RNA gene amplicon sequences. The number of chiggers and the percent identity are included in parentheses after each species.

| **Collection sites** | **Number of chiggers** | **Chigger identification results** | **Ecoregion** |
| --- | --- | --- | --- |
| Lake James State Park (LJSP) | 7 | *Pseudoschoengastia* sp. (n=1, 96.04%), *Eutrombicula splendens* (n=5, 100%), *Eutrombicula tinami* (n=1, 100%) | Mountains |
| Morrow Mountain State Park (MMSP) | 9 | *Pseudoschoengastia* sp. (n=2, 96.01-96.3%), *Eutrombicula splendens* (n=7, 100%) | Piedmont |
| Pee Dee National Wildlife Refuge (PDNWR) | 9 | *Eutrombicula splendens* (n=7, 99.42-100%), *Eutrombicula tinami* (n=2, 100%) | Piedmont |
| Jordan Lake State Recreation Area (JLSRA) | 6 | *Eutrombicula splendens* (n=6, 99.7-100%) | Piedmont |
| William B. Umstead State Park (WBUSP) | 8 | *Pseudoschoengastia* sp. (n=3, 96.02-96.05%), *Eutrombicula splendens* (n=5, 99.77-100%) | Piedmont |
| Falls Lake State Recreation Area (FLSRA) | 7 | *Pseudoschoengastia* sp. (n=2, 95.86-96.03%), *Eutrombicula splendens* (n=4, 100%), *Eutrombicula tinami* (n=1, 100%) | Piedmont |
| Kerr Lake State Recreation Area (KLSRA) | 8 | *Pseudoschoengastia* sp. (n=1, 96.07%), *Eutrombicula splendens* (n=7, 99.77-100%) | Piedmont |
| CNF (Croatan National Forest) | 8 | *Pseudoschoengastia* sp. (n=1, 95.2%), *Eutrombicula splendens* (n=7, 100%) | Coastal plain |
| Lumber River State Park (LRSP) | 7 | *Pseudoschoengastia* sp (n=1, 96.10%), *Eutrombicula splendens* (n=6, 99-100%) | Coastal plain |
