# Supplementary material for: Geographic and Orientia infection status influence on the bacterial microbiome of free-living chiggers in North Carolina, USA
Source: PLoS One. 2026 Jul 8;21(7):e0353174. doi: 10.1371/journal.pone.0353174 (PMC13345271; doi:10.1371/journal.pone.0353174)
Supplement: S2 Table — LJSP, Lake James State Park; MMSP, Morrow Mountain State Park; PDNWR, Pee Dee National Wildlife Refuge; LRSP, Lumber River State Park; JLSRA, Jordan Lake State Recreational Area; WBUSP, William B. Umstead State Park; FLSRA, Falls Lake State Recreational Area; KLSRA, Kerr Lake State Recreational Area; CNF, Croatan National Forest. (DOCX) [file pone.0353174.s002.docx]

**Table S2**. Pairwise PERMANOVA analysis results for free-living chiggers, *Eutrombicula splendens*, collected from nine different locations based on the weighted UniFrac. LJSP, Lake James State Park; MMSP, Morrow Mountain State Park; PDNWR, Pee Dee National Wildlife Refuge; LRSP, Lumber River State Park; JLSRA, Jordan Lake State Recreational Area; WBUSP, William B. Umstead State Park; FLSRA, Falls Lake State Recreational Area; KLSRA, Kerr Lake State Recreational Area; CNF, Croatan National Forest.

| **Group 1** | **Group 2** | **Sample Size** | ***p*-value** |
| --- | --- | --- | --- |
| LJSP | MMSP·· | 12 | 0.688 |
|  | PDNWR | 12 | 0.281 |
|  | LRSP | 11 | 0.007 |
|  | JLSRA | 11 | 0.005 |
|  | WBUSP | 10 | 0.048 |
|  | FLSRA | 9 | 0.007 |
|  | KLSRA | 12 | 0.003 |
|  | CNF | 12 | 0.101 |
| MMSP | PDNWR | 14 | 0.136 |
|  | LRSP | 13 | 0.024 |
|  | JLSRA | 13 | 0.007 |
|  | WBUSP | 12 | 0.175 |
|  | FLSRA | 11 | 0.068 |
|  | KLSRA | 14 | 0.001 |
|  | CNF | 14 | 0.265 |
| PDNWR | LRSP | 13 | 0.014 |
|  | JLSRA | 13 | 0.012 |
|  | WBUSP | 12 | 0.047 |
|  | FLSRA | 11 | 0.045 |
|  | KLSRA | 14 | 0.001 |
|  | CNF | 14 | 0.002 |
| LRSP | JLSRA | 12 | 0.260 |
|  | WBUSP | 11 | 0.163 |
|  | FLSRA | 10 | 0.225 |
|  | KLSRA | 13 | 0.035 |
|  | CNF | 13 | 0.009 |
| JLSRA | WBUSP | 11 | 0.212 |
|  | FLSRA | 10 | 0.565 |
|  | KLSRA | 13 | 0.001 |
|  | CNF | 13 | 0.001 |
| WBUSP | FLSRA | 9 | 0.478 |
|  | KLSRA | 12 | 0.002 |
|  | CNF | 12 | 0.007 |
| FLSRA | KLSRA | 11 | 0.005 |
|  | CNF | 11 | 0.004 |
| KLSRA | CNF | 14 | 0.001 |
