# Supplementary figures and images for: Geographic and Orientia infection status influence on the bacterial microbiome of free-living chiggers in North Carolina, USA
Source: PLoS One. 2026 Jul 8;21(7):e0353174. doi: 10.1371/journal.pone.0353174 (PMC13345271; doi:10.1371/journal.pone.0353174)

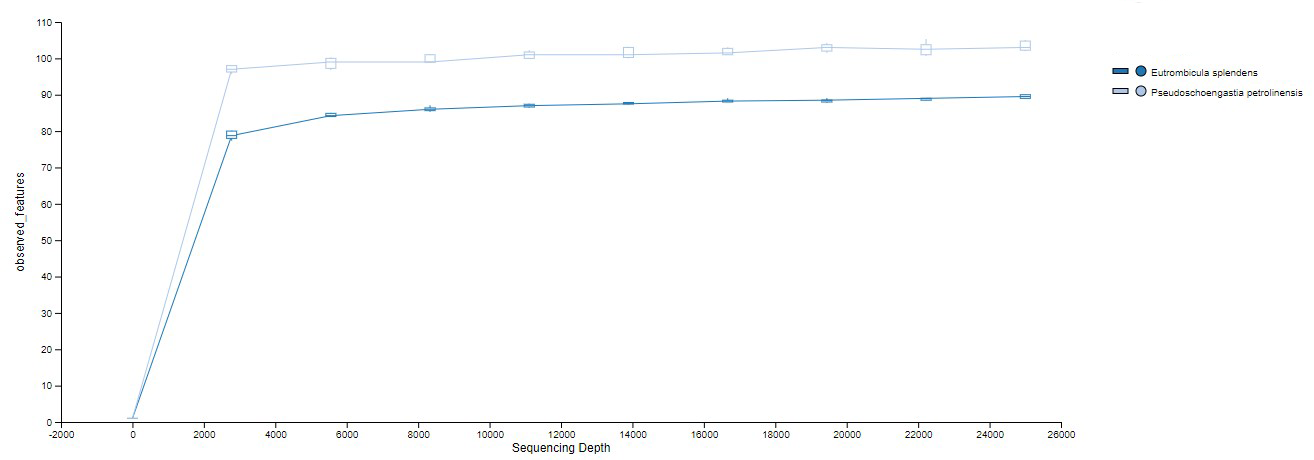

Supplement: S1 Fig — Error bars represent the standard error of the mean. (TIF) [file pone.0353174.s003.tif]

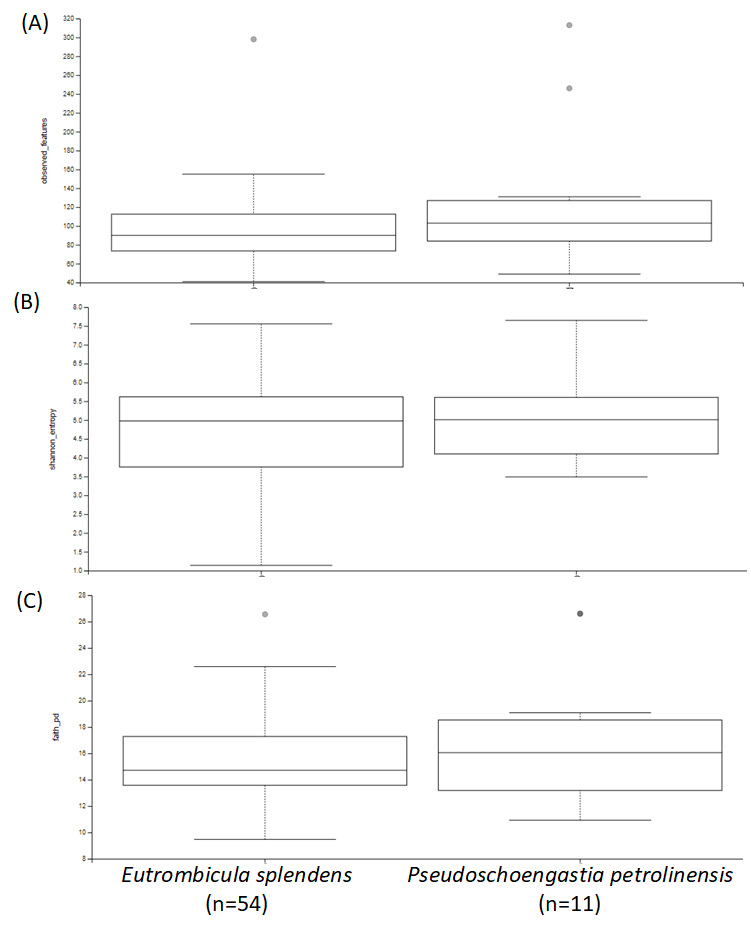

Supplement: S2 Fig — (A) Observed ASVs, Shannon diversity, (B) Shannon diversity, and (C) Faiths phylogenetic diversity. (TIF) [file pone.0353174.s004.tif]

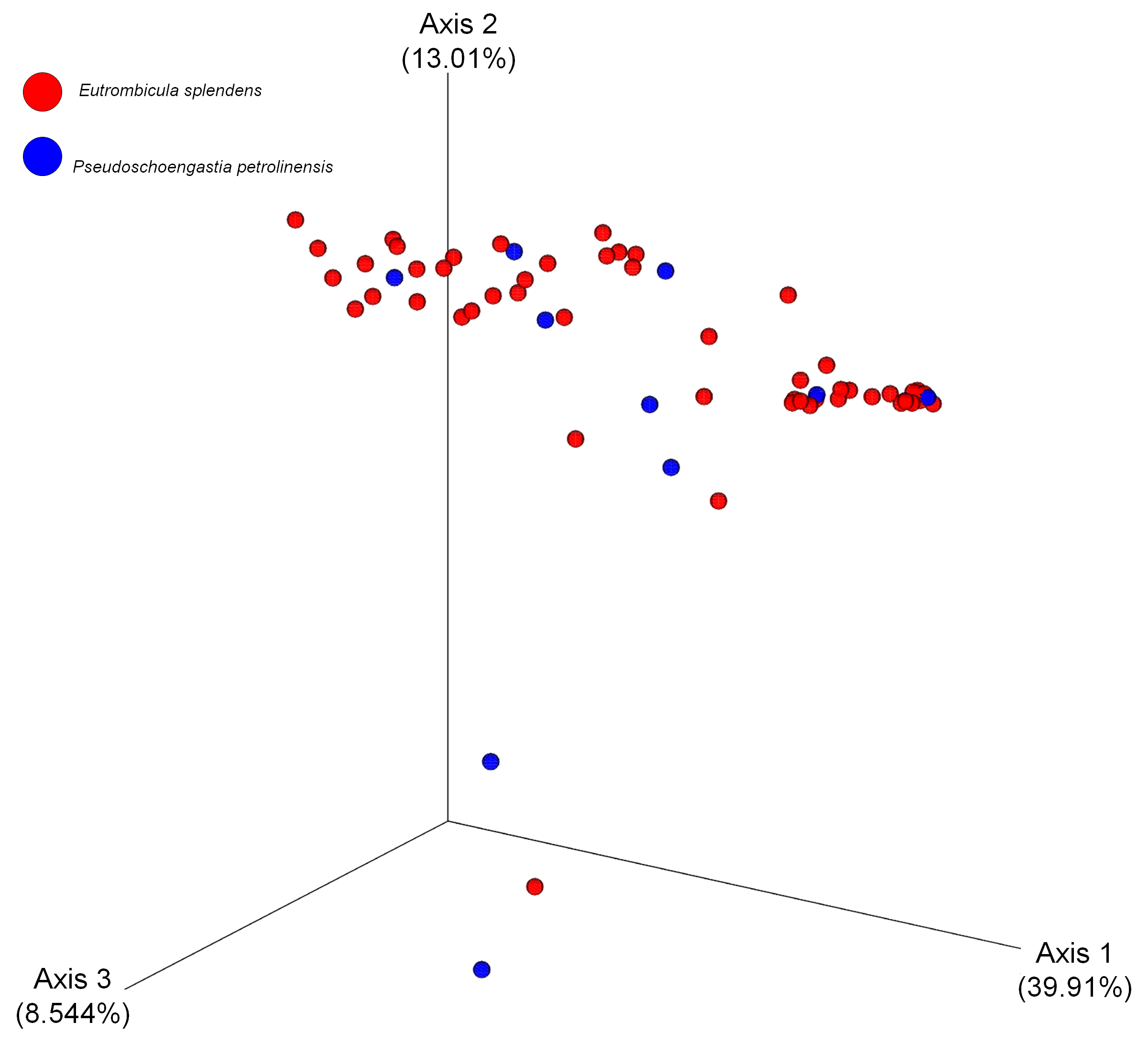

Supplement: S3 Fig — Analysis was based on the weighted Unifrac metric. (TIF) [file pone.0353174.s005.tif]

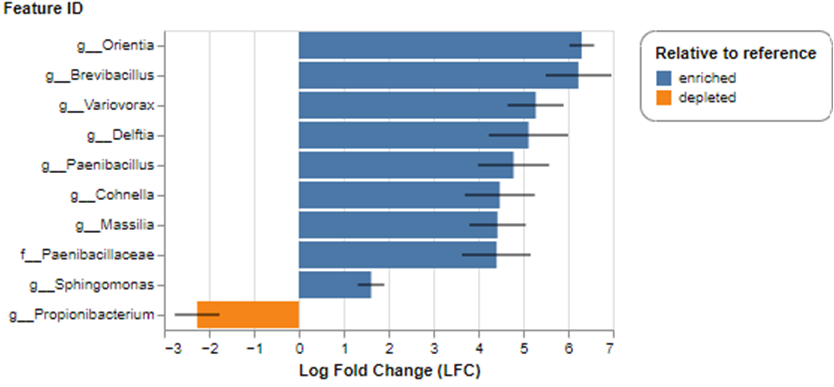

Supplement: S4 Fig — (TIF) [file pone.0353174.s006.tif]

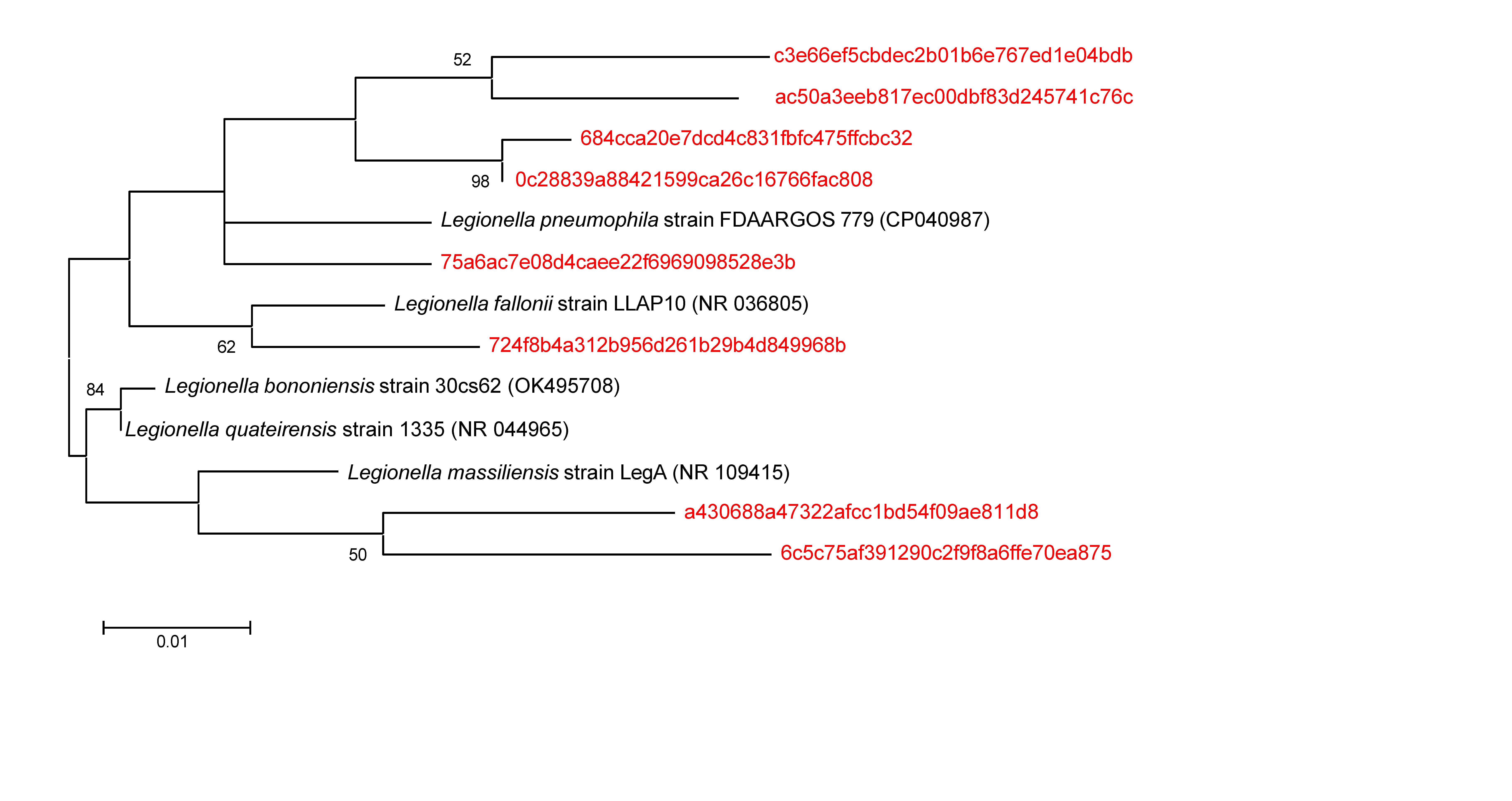

Supplement: S7 Fig — Phylogenetic tree constructed by the Maximum-likelihood (ML) method based on 16S rRNA gene. (TIF) [file pone.0353174.s009.tif]
