# Supplementary material for: Geographic and Orientia infection status influence on the bacterial microbiome of free-living chiggers in North Carolina, USA
Source: PLoS One. 2026 Jul 8;21(7):e0353174. doi: 10.1371/journal.pone.0353174 (PMC13345271; doi:10.1371/journal.pone.0353174)
Supplement: Attachment — Submitted filename: Comments to the Author.pdf [file pone.0353174.s012.pdf]

Reviewer Comments

Manuscript Number: PONE-D-25-67871

Title: Geographic and Orientia infection status influence on the bacterial microbiome of freeliving chiggers in North Carolina, USA

Journal: PLOS One

Comments to the Author

This study investigates the bacterial microbiome of free-living chiggers in North Carolina, USA, and explores its associations with geographic factors and *Orientia* infection. While the research question is relevant, the manuscript contains fundamental methodological deficiencies. These deficiencies primarily concern the identification of free-living chigger specimens, the description of sampling background, and the confirmation of life-history stages.

Major Comments (must be revised or clearly explained)

1. Inadequate identification of free-living chiggers and absence of morphological evidence

The internationally accepted research framework for chiggers (Trombiculidae) is based on the examination of parasitic larvae collected directly from the body surface of animal hosts. Chiggers parasitize small mammals exclusively at the larval stage, during which they feed on host blood. After feeding, larvae detach from the host and subsequently develop into nymphs and adults. These later stages are free-living and non-parasitic. Therefore, the ecological condition of being "free-living" cannot be directly equated with chigger larvae, nor can it be used as evidence of vector competence.

(1) Absence of field recognition and morphological identification

The manuscript reports the collection of "red moving objects (<250 μm)" observed on black tiles. This description does not constitute a diagnostic characteristic of chiggers. Numerous soil microarthropods, including other mites and immature insects, may fall within this size range and display similar coloration. The authors must clarify whether voucher specimens were slide-mounted and preserved. It is also necessary to specify whether all samples, or only a subset, were examined microscopically prior to molecular analysis, including examination under oil immersion when appropriate.

Furthermore, the authors must state whether non-chigger arthropods were excluded based on

fundamental larval chigger characters, such as body morphology, leg number and segmentation, and the presence and structure of the scutum. The manuscript should clearly indicate which diagnostic characters were used and which taxonomic keys or references were followed. If no morphological identification was conducted, this omission represents a serious methodological limitation. In that case, the authors must explicitly acknowledge this limitation in the Discussion and evaluate its potential impact on the study's conclusions.

(2) Lack of confirmation of life-history stage

Only the larval stage of chiggers is parasitic and of medical relevance. The manuscript refers to the collected specimens as "free-living, unfed chiggers," yet provides no explanation of how the authors confirmed that all specimens were larvae rather than unfed nymphs or adults. Larvae possess three pairs of legs, whereas nymphs and adults possess four pairs, a clear and diagnostic distinction. No morphological evidence is presented to confirm life-history stage. If non-larval stages were included, then the fundamental assumption regarding disease transmission potential is invalid.

2. Unclear description of DNA extraction and library preparation from single chiggers

In the section "DNA extraction and chigger identification," the authors state that "individual, free-living chiggers were surface sterilized," implying that DNA extraction was performed on single specimens. This raises significant technical concerns. Individual chiggers are extremely small and possess very low biomass. Obtaining sufficient DNA from a single unfed chigger larva for both high-quality 16S rRNA amplicon sequencing and 18S rRNA Sanger sequencing is technically challenging and often associated with a high failure rate.

The authors must clearly state whether each "individual" sample represents a single chigger or a pooled sample consisting of multiple chiggers collected from the same site or batch. This distinction is critical, as it determines the biological unit of downstream analyses and whether the results represent individual-level microbiomes or pooled community profiles. The authors must also report DNA extraction efficiency and PCR amplification success rates, and specify whether any samples were excluded due to insufficient DNA quantity. These details are essential for evaluating potential sample selection bias and ensuring reproducibility.

3. Inadequate description and absence of key parameters in the molecular species identification method

(1) Gene choice and similarity threshold

The use of the 18S rRNA gene for chigger species identification is acceptable. However, the manuscript does not report the sequence similarity threshold applied in BLASTn comparisons. This parameter is central to molecular taxonomic assignment and determines whether identification at the genus or species level is justified.

(2) Specific concern regarding sequence similarity

In the Results section (page 13), the manuscript reports that *P. petrolinensis* sequences show 95.2–96.3% similarity to reference sequences. The authors must specify the identification threshold used and justify its application. Similarity values in this range are generally insufficient for confident species-level identification and more commonly support genus-level assignment or suggest the presence of cryptic or undescribed taxa. The authors must explain why these sequences were assigned to *P. petrolinensis*, and clarify whether morphological evidence, similarity overlap among related taxa, or phylogenetic analyses were used to support this conclusion. The criteria for species determination must be explicitly stated.

4. Insufficient description of sampling locations and ecological background

Although several nature reserves in North Carolina are listed as sampling sites, the manuscript does not adequately describe the specific microhabitats in which mites were collected. It is also unclear whether the sampling locations were associated with small mammal activity or suitable host habitats, or whether potential hosts were present in the vicinity.

Under the current description, the collection of free-moving mites from surface environments alone is insufficient to establish that the specimens are chigger larvae with prior parasitic history or vector potential.

Recommendations for Revision

If the identity of the study organisms as chigger larvae has not been reliably confirmed, then all subsequent inferences are based on an unstable biological premise. The manuscript therefore exhibits a high risk of over-interpretation. In a revised version, the authors must address at least the following points:

1. Provide systematic morphological identification evidence and clearly confirm the life-history stage of all specimens.

2. Explicitly state the similarity thresholds and decision criteria used for 18S rRNA-based

molecular identification.

3. In the DNA extraction section, clearly define what constitutes a single sample—whether it is an individual chigger mite or a pooled sample of chigger mites. Include detailed extraction procedures, as well as the success rates of extraction and PCR.

4. If larval identity cannot be confirmed, use the term “chiggers” with caution throughout the manuscript and fully discuss this limitation.
