# Supplementary material for: Geographic and Orientia infection status influence on the bacterial microbiome of free-living chiggers in North Carolina, USA
Source: PLoS One. 2026 Jul 8;21(7):e0353174. doi: 10.1371/journal.pone.0353174 (PMC13345271; doi:10.1371/journal.pone.0353174)
Supplement: Attachment — Submitted filename: Comments and response to Reviewer #1and2_Final.docx [file pone.0353174.s014.docx]

**Reviewer #1**: Comments to the Author
This study investigates the bacterial microbiome of free-living chiggers in North Carolina, USA, and explores its associations with geographic factors and Orientia infection. While the research question is relevant, the manuscript contains fundamental methodological deficiencies. These deficiencies primarily concern the identification of free-living chigger specimens, the description of sampling background, and the confirmation of life-history stages.

**Authors’ response: We thank the reviewer for the careful evaluation of our manuscript and for recognizing the relevance of the research question, particularly regarding chigger specimen identification and confirmation of life-history stages. Detailed replies to the comments are given below.**

Major Comments (must be revised or clearly explained)
1. Inadequate identification of free-living chiggers and absence of morphological evidence
The internationally accepted research framework for chiggers (Trombiculidae) is based on the examination of parasitic larvae collected directly from the body surface of animal hosts. Chiggers parasitize small mammals exclusively at the larval stage, during which they feed on host blood. After feeding, larvae detach from the host and subsequently develop into nymphs and adults. These later stages are free-living and non-parasitic. Therefore, the ecological condition of being “free-living” cannot be directly equated with chigger larvae, nor can it be used as evidence of vector competence.

**Authors’ response: We appreciate the reviewer’s critical distinction between the parasitic larval stage and the free-living post-larval stages (nymphs and adults) of Trombiculidae, and we agree that vector competence is exclusive to the larvae. To clarify, our use of the term “free-living” referred specifically to the collection site (unattached larvae questing in soil and vegetation) rather than their biological stage. To address the reviewer’s concern regarding identification and morphological evidence, all collected specimens were examined under a stereo microscope to confirm they were in the hexapod (six-legged) larval stage, distinguishing them from the octopod (eight-legged) nymphs and adults. Furthermore, to ensure taxonomic accuracy, representative sub-samples from these collections were validated by Dr. Dac Crossley (Curator of Acari at the Georgia Museum of Natural History, Georgia, USA) a specialist in Trombiculid taxonomy, to ensure the stage and species-level identification. We have updated the Methods section to explicitly state these morphological criteria and our external validation process.**

(1) Absence of field recognition and morphological identification
The manuscript reports the collection of “red moving objects (<250 μm)” observed on black tiles. This description does not constitute a diagnostic characteristic of chiggers. Numerous soil microarthropods, including other mites and immature insects, may fall within this size range and display similar coloration. The authors must clarify whether voucher specimens were slide-mounted and preserved. It is also necessary to specify whether all samples, or only a subset, were examined microscopically prior to molecular analysis, including examination under oil immersion when appropriate.
Furthermore, the authors must state whether non-chigger arthropods were excluded based on fundamental larval chigger characters, such as body morphology, leg number and segmentation, and the presence and structure of the scutum. The manuscript should clearly indicate which diagnostic characters were used and which taxonomic keys or references were followed. If no morphological identification was conducted, this omission represents a serious methodological limitation. In that case, the authors must explicitly acknowledge this limitation in the Discussion and evaluate its potential impact on the study’s conclusions.

**Authors’ Response: We agree with the reviewer that the field observation of "red moving objects (<250 μm)" on black tiles is not diagnostic of chiggers and could apply to various soil microarthropods. This description was intended only to reflect the initial field observation and was not the primary basis for identification.**

**Following collection, specimens were transported to the laboratory and examined under a stereomicroscope. Identification as larval chiggers (family Trombiculidae) was confirmed based on established diagnostic criteria, including, the presence of six legs (distinguishing larvae from nymphal and adult mites), overall body morphology consistent with trombiculid larvae, and characteristic leg segmentation as described in taxonomic keys (Wharton et al., 1952; Brennan et al., 1959; Bennett et al., 2014). These features were used to exclude non-chigger arthropods collected during environmental sampling.**

**To ensure accuracy of life stage and species identification, randomly selected subsamples from each collection site were submitted to Dr. Dac Crossley, Curator of Acari at the Georgia Museum of Natural History, for independent life-stage and morphological validation. Representative specimens from each collection batch were slide-mounted, preserved as vouchers, and examined via compound microscopy (including oil-immersion where necessary). Identification was performed using standard taxonomic keys (Wharton et al., 1952; Brennan et al., 1959; Bennett et al., 2014).**

**Because slide-mounting permanently alters specimens, detailed morphological examination was conducted on representative subsamples rather than the entire collection. All remaining specimens were subsequently validated using molecular methods prior to downstream analyses. The Methods section has been revised to explicitly detail the morphological examination procedures, the subsampling strategy, and the external taxonomic validation.**

(2) Lack of confirmation of life-history stage
Only the larval stage of chiggers is parasitic and of medical relevance. The manuscript refers to the collected specimens as “free-living, unfed chiggers,” yet provides no explanation of how the authors confirmed that all specimens were larvae rather than unfed nymphs or adults. Larvae possess three pairs of legs, whereas nymphs and adults possess four pairs, a clear and diagnostic distinction. No morphological evidence is presented to confirm life-history stage. If non-larval stages were included, then the fundamental assumption regarding disease transmission potential is invalid.

**Authors’ response: As we stated in our response above, the collected samples were examined after collection to confirm that they were in the larval stage of mites. We acknowledge that these essential identification steps were not described in sufficient detail in the original manuscript, which may have caused confusion regarding how the life-history stage was verified. This level of “due diligence” to identify specimens is standard practice for our group and applied also to our previously published papers on our work with chiggers in the US and their infection with *Orientia* spp. bacteria.**

2. Unclear description of DNA extraction and library preparation from single chiggers
In the section “DNA extraction and chigger identification,” the authors state that “individual, free-living chiggers were surface sterilized,” implying that DNA extraction was performed on single specimens. This raises significant technical concerns. Individual chiggers are extremely small and possess very low biomass. Obtaining sufficient DNA from a single unfed chigger larva for both high-quality 16S rRNA amplicon sequencing and 18S rRNA Sanger sequencing is technically challenging and often associated with a high failure rate. The authors must clearly state whether each “individual” sample represents a single chigger or a pooled sample consisting of multiple chiggers collected from the same site or batch. This distinction is critical, as it determines the biological unit of downstream analyses and whether the results represent individual-level microbiomes or pooled community profiles. The authors must also report DNA extraction efficiency and PCR amplification success rates, and specify whether any samples were excluded due to insufficient DNA quantity. These details are essential for evaluating potential sample selection bias and ensuring reproducibility.

**Authors’ response: We confirm that DNA extraction was performed on individual chigger larvae; each “individual” sample reported in the manuscript represents a single chigger rather than a pooled specimen. Initially, 120 chiggers were processed separately for DNA extraction. To maximize DNA recovery and concentration from these single specimens, we optimized the extraction protocol by reducing the final elution volume to 50 μL (instead of the manufacturer’s recommended 200 μL). This modification ensured sufficient DNA concentration for downstream PCR applications. Furthermore, advances in molecular technology now allow for the detection of DNA from extremely small biological samples, even from a single hair, supporting the feasibility and reliability of single-specimen DNA analysis.**

**Following extraction, NanoDrop spectrophotometry was used as an initial quality control step. Twenty-five samples did not yield detectable DNA and were therefore excluded from further analysis. The remaining 95 individual samples were subjected to 16S rRNA microbiome analysis, as described in Chen et al. (2023). As noted in Chen et al. (2023), chigger species identification was performed using both morphological and molecular approaches on subsampled material. For the present study, we conducted 18S rRNA gene amplification and Sanger sequencing on all 95 samples to enable species-level identification and to assess microbiome variation among species. Although 18S amplification was successful for all samples, 26 produced ambiguous sequencing results. Consequently, 69 samples yielded high-quality sequences suitable for identification, while the 26 ambiguous samples were excluded from the final analysis. These methodological details and the technical limitations inherent to single-chigger DNA extraction have now been clarified and incorporated into the revised manuscript.**

3. Inadequate description and absence of key parameters in the molecular species identification method
(1) Gene choice and similarity threshold
The use of the 18S rRNA gene for chigger species identification is acceptable. However, the manuscript does not report the sequence similarity threshold applied in BLASTn comparisons. This parameter is central to molecular taxonomic assignment and determines whether identification at the genus or species level is justified.

**Authors’ response: We agree that this was not explained in the earlier version of the manuscript. In the revised manuscript, we have now explicitly described the criteria applied for BLASTn-based identification using the 18S rRNA gene.**

(2) Specific concern regarding sequence similarity
In the Results section (page 13), the manuscript reports that P. petrolinensis sequences show 95.2–96.3% similarity to reference sequences. The authors must specify the identification threshold used and justify its application. Similarity values in this range are generally insufficient for confident species-level identification and more commonly support genus-level assignment or suggest the presence of cryptic or undescribed taxa. The authors must explain why these sequences were assigned to P. petrolinensis, and clarify whether morphological evidence, similarity overlap among related taxa, or phylogenetic analyses were used to support this conclusion. The criteria for species determination must be explicitly stated.

**Authors’ response: We acknowledge that the observed sequence similarity of 95.2–96.3% falls below the commonly accepted 97–99% threshold for confident species-level identification in many taxa. Therefore, we have revised the classification to the genus *Pseudoschoengastia*, consistent with the supporting morphological evidence. The manuscript has been updated accordingly, and the rationale for this change is now clearly explained in the revised version.**

4. Insufficient description of sampling locations and ecological background
Although several nature reserves in North Carolina are listed as sampling sites, the manuscript does not adequately describe the specific microhabitats in which mites were collected. It is also unclear whether the sampling locations were associated with small mammal activity or suitable host habitats, or whether potential hosts were present in the vicinity. Under the current description, the collection of free-moving mites from surface environments alone is insufficient to establish that the specimens are chigger larvae with prior parasitic history or vector potential.

**Authors’ response: We sincerely thank the reviewer for this valuable comment. Unfortunately, detailed ecological data were not collected at the time of sampling. Specifically, we did not record microhabitat characteristics, small mammal activity, host presence, or vegetation type at the collection sites. In addition, chiggers were collected exclusively from surface environments and were not obtained directly from vertebrate hosts.**

Recommendations for Revision
If the identity of the study organisms as chigger larvae has not been reliably confirmed, then all subsequent inferences are based on an unstable biological premise. The manuscript therefore exhibits a high risk of over-interpretation. In a revised version, the authors must address at least the following points:

1. Provide systematic morphological identification evidence and clearly confirm the life-history stage of all specimens.

**Authors’ response: We confirm that all specimens were subjected to systematic morphological examination using standard taxonomic keys and diagnostic characters specific to this group. The distinguishing morphological features used for identification are now clearly described in the revised manuscript to ensure transparency in taxonomic assignment. All specimens analyzed in this study were confirmed to be in the larval stage based on characteristic morphological traits (e.g., number of legs, body segmentation). This clarification has been added to the Methods section to eliminate any ambiguity regarding the life-history stage of the analyzed specimens.**

2. Explicitly state the similarity thresholds and decision criteria used for 18S rRNA-based molecular identification.

**Authors’ response: We agree that the decision criteria for 18S rRNA–based identification should be explicitly stated. This concern has been addressed in the revised manuscript, where we now detail the thresholds and the decision framework used for taxonomic assignments.**

3. In the DNA extraction section, clearly define what constitutes a single sample—whether it is an individual chigger mite or a pooled sample of chigger mites. Include detailed extraction procedures, as well as the success rates of extraction and PCR.

**Authors’ response: We can confirm that each sample consisted of a single, individual chigger mite rather than a pooled sample. As the reviewer’s suggested, we have also added a detailed step-by-step extraction procedure and included the specific success rates for both the DNA extraction and the subsequent PCR amplification.**

4. If larval identity cannot be confirmed, use the term “chiggers” with caution throughout the manuscript and fully discuss this limitation.

**Authors’ response: In the revised manuscript, we have clarified in the methodology that the larval identity was confirmed as chiggers based on the presence of six legs. Because our morphological identification was definitive, we believe the term 'chiggers' is used correctly. However, as requested, we have added a brief statement in the Methodology and Discussion sections to provide the specific morphological criteria used for this confirmation.**

Reviewer #2:

The manuscript "Geographic and Orientia infection status influence on the bacterial microbiome of freeliving chiggers in North Carolina, USA" describes sampling of 3 different species of chiggers from 3 different ecological region in the state of North Carolina in the US, and a subsequent characterization of their bacterial microbiome using 16S amplicon sequencing. Fleas and chiggers are historically known vectors of various infectious diseases, but are also emerging as potential vectors of new pathogens in new geographical areas. Characterizing their geographical distribution as well as their community of microbes is important for both ecological studies as well as public health reasons. This well-written manuscript is a welcome addition to this area of research. The overall experimental design is appropriate, experiments are well executed, and data analysis and presentation is of good quality. The details of experimental methods and data analysis is described in reasonable detail. The raw data seem to have been deposited in the public NCBI SRA database under bioproject PRJNA1285095 and SUB15424311. which will hopefully be released publicly upon acceptance/publication of the manuscript. There are only 3 major a few minor points that the authors need to address before this manuscript can be accepted for publication.
**Authors’ response: Thank you for taking the time to review our manuscript, and we sincerely appreciate the reviewer’s positive comments. The sequencing data were submitted under bioproject PRJNA1285095 and SUB15424311 and will be publicly accessible after the release date of March 31, 2026.**

Major points:
1. In the methods section, the authors have not specified the Illumina instrument used for sequencing. This is critical information, as it influences the choice of denoising algorithm. Starting with NovaSeq instruments, Illumina started binning per-base quality scores instead of a monotonic continuous scale. However, the DADA2 pipeline assumes a continuous scoring scheme and is therefore not suitable for FASTQs produced by NovaSeq or later instruments. If the authors used an Illumina instrument which compresses FASTQ quality scores into 4 or 5 bins (e.g. NovaSeq), DADA2 cannot be used and Deblur is recommended instead. For more discussions see <https://github.com/benjjneb/dada2/issues/791> and <https://forum.qiime2.org/t/novaseq-and-dada2-incompatibility/25865> . Please provide instrument details and choose Deblur over DADA2 if needed.

**Authors’ response: Thank you for this important comment. Sequencing was performed on Illumina MiSeq platform. MiSeq generates continuous quality scores and does not use quality score binning. Therefore, DADA2 is appropriate for this dataset. We have now added the instrument details to the Methods section.**

2. The Greengenes database has been superseded by Greengenes2 for quite some time now, with obvious improvements in taxonomical breadth and accuracy. As per their methods, the authors have used "Greengenes 13_8" which was last updated in 2013 (<https://ftp.microbio.me/greengenes_release/>). Please use the latest GreenGenes2 database in all analysis. For more discussion see <https://forum.qiime2.org/t/greengenes2-2024-09/31606>. Also, update the citation 40 from GreenGenes to Greengenes2, described in McDonald et al. Nature Biotechnology (2023) : <https://www.nature.com/articles/s41587-023-01845-1>

**Authors’ response: Thank you for this important suggestion. As recommended by the reviewer, we have reclassified all ASVs using the latest Greengenes2 database (v. 2024.09). Figures 8 and 9, the corresponding text, as well as reference 40 (new number is 43) have been updated accordingly.**

3. In Results, starting line 248 onwards, the authors have described the taxa observed in the subset ASVs that *could* be classified ("32 bacterial phyla, 87 classes, 158 orders, 282 families, and 521 genera"). However, it is not clear how many of the 3,575 ASVs could be classified into these levels versus what fraction of ASVs remained unclassified. Please provide these proportions at all taxonomic levels. It is not uncommon for more than 70% of the ASVs to remain unclassified, especially for under-studied microbiomes such the one these authors are studying - but discussing only the classifiable fraction creates to a false impression that there is no more bacterial diversity remaining to be discovered in these microbiomes!

**Authors’ response: Thank you for this important comment. To address this concern, we reclassified all ASVs using Greengenes2 and have added the updated proportions of ASVs classified at the phylum, class, order, family, and genus levels in the Results section.**

Minor points:
1. Median values are a more robust statistical descriptors of distribution of data than means. Hence it is recommended that the authors report median values wherever possible e.g. line 246 "average of 94,989 reads per sample", line 351 onwards multiple instances of "mean relative abundance".

**Authors’ response: As suggested, we have revised the manuscript to report the median sequencing depth and median relative abundances.**

2. Line 247: "After filtering ASVs based on total abundance and prevalence in the
dataset" : What were the criteria/cut-offs used in this filtering step?

**Authors’ response: Thank you for pointing this out. No additional filtering based on total abundance or prevalence was applied. After quality filtering and denoising using the DADA2 plugin, a total of 6,554,232 high-quality reads were retained, from which 3,575 amplicon sequence variants (ASVs) were inferred and used for all downstream analyses. The sentence in the manuscript has been revised accordingly.**

3. Line 460 onwards in Discussion: The authors find the absence of difference in alpha diversity measures between different host chigger species. However, alpha diversity is a within sample measurement. Thus it is technically possible for two microbiomes to be composed of different species (represented by different unique ASV sequences) but have the same diversity metrics (same *number* of unique ASVs). Here is a very simplified toy example to show this: chigger-species1 has 3 microbes A, B and C while chigger-species2 has three different microbes D, E, and F - such that the diversity metric is 3 for both chigger-species.
Did the authors check if indeed the same set of ASVs were found in all three chigger species? Please also report the median number of ASVs observed per sample (not all 3,575 ASVs are expected to be seen in each sample).

**Authors’ response: Thank you for the comments. We agree that samples with same alpha diversity metrics do not mean they have identical community composition. To avoid confusion, we have added the beta diversity results in the discussion. No significant difference was found between *E. splendens* and *P. petrolinensis* (now revised to the genus *Pseudoschoengastia*)(PERMANOVA, p = 0.125). We have calculated the median number of observed ASVs per sample based on observed features of alpha diversity analysis. The median number of ASVs per sample was 91. Corresponding text has been updated.**

3. In Figures 2 and 3, "Observed fatures" could be changed to "Observed ASVs"?

**Authors’ response: The change has been made as suggested.**

4. The reference 30 cites a PhD thesis, which I believe has now been published as a peer-reviewed research article: Alkathiry et al. Microbiome and mitogenomics of the chigger mite Pentidionis agamae: potential role as an Orientia vector and associations with divergent clades of Wolbachia and Borrelia. BMC Genomics (2024) at <https://doi.org/10.1186/s12864-024-10301-6> . Please update accordingly.

**Authors’ response: As recommended, we have updated Reference 30 to cite the published peer-reviewed article by Alkathiry et al. (2024, BMC Genomics) instead of the PhD thesis. The reference list has been revised accordingly..**
