# Supplementary material for: Geographic and Orientia infection status influence on the bacterial microbiome of free-living chiggers in North Carolina, USA
Source: PLoS One. 2026 Jul 8;21(7):e0353174. doi: 10.1371/journal.pone.0353174 (PMC13345271; doi:10.1371/journal.pone.0353174)
Supplement: Peer review history

# Peer review: Decision Letter 0

**DOI:** 10.1371/journal.pone.0353174.r001

**Authors:** Yong Qi

25 Jan 2026

PONE-D-25-67871

Geographic and Orientia infection status influence on the bacterial microbiome of free-living chiggers in North Carolina, USA

PLOS One

Dear Dr. Ponnusamy,

Thank you for submitting your manuscript to PLOS ONE. After careful consideration, we feel that it has merit but does not fully meet PLOS ONE’s publication criteria as it currently stands. Therefore, we invite you to submit a revised version of the manuscript that addresses the points raised during the review process.

Please submit your revised manuscript by Mar 11 2026 11:59PM. If you will need more time than this to complete your revisions, please reply to this message or contact the journal office at plosone@plos.org. When you're ready to submit your revision, log on to https://www.editorialmanager.com/pone/ and select the 'Submissions Needing Revision' folder to locate your manuscript file.

Please include the following items when submitting your revised manuscript:

- A letter that responds to each point raised by the academic editor and reviewer(s). You should upload this letter as a separate file labeled 'Response to Reviewers'.
- A marked-up copy of your manuscript that highlights changes made to the original version. You should upload this as a separate file labeled 'Revised Manuscript with Track Changes'.
- An unmarked version of your revised paper without tracked changes. You should upload this as a separate file labeled 'Manuscript'.

If you would like to make changes to your financial disclosure, please include your updated statement in your cover letter. Guidelines for resubmitting your figure files are available below the reviewer comments at the end of this letter.

If applicable, we recommend that you deposit your laboratory protocols in protocols.io to enhance the reproducibility of your results. Protocols.io assigns your protocol its own identifier (DOI) so that it can be cited independently in the future. For instructions see: https://journals.plos.org/plosone/s/submission-guidelines#loc-laboratory-protocols. Additionally, PLOS ONE offers an option for publishing peer-reviewed Lab Protocol articles, which describe protocols hosted on protocols.io. Read more information on sharing protocols at https://plos.org/protocols?utm_medium=editorial-email&utm_source=authorletters&utm_campaign=protocols.

We look forward to receiving your revised manuscript.

Kind regards,

Yong Qi

Academic Editor

PLOS One

Journal Requirements:

When submitting your revision, we need you to address these additional requirements.

1. Please ensure that your manuscript meets PLOS ONE's style requirements, including those for file naming. The PLOS ONE style templates can be found at

https://journals.plos.org/plosone/s/file?id=wjVg/PLOSOne_formatting_sample_main_body.pdf and

https://journals.plos.org/plosone/s/file?id=ba62/PLOSOne_formatting_sample_title_authors_affiliations.pdf

2. In your Methods section, please provide additional information regarding the permits you obtained for the work. Please ensure you have included the full name of the authority that approved the field site access and, if no permits were required, a brief statement explaining why.

3. Thank you for stating the following in the Acknowledgments Section of your manuscript:

“The study was financially supported by the National Institutes of Health, National Institute of Allergy and Infectious Diseases (grant no. 1R03AI166406-01) and the Southeast Center for Agricultural Health and Injury Prevention. The research was also funded by the Department of the Army, US Army Contracting Command, Aberdeen Proving Ground, Edgewood Contracting Division, Ft Detrick MD, Deployed Warfighter Protection (DWFP) Program Grant W911QY1910003 and E911SR2210005. The work was also supported by the Research Capacity Fund (HATCH), project award no. 02853, from the U.S. Department of Agriculture’s National Institute of Food and Agriculture. Any opinions, findings, conclusions, or recommendations expressed in this publication are those of the authors and should not be construed to represent any official USDA or U.S. Government determination or policy.”

We note that you have provided funding information that is not currently declared in your Funding Statement. However, funding information should not appear in the Acknowledgments section or other areas of your manuscript. We will only publish funding information present in the Funding Statement section of the online submission form.

Please remove any funding-related text from the manuscript and let us know how you would like to update your Funding Statement. Currently, your Funding Statement reads as follows:

“The study was financially supported by the National Institutes of Health, National Institute of Allergy and Infectious Diseases (grant no. 1R03AI166406-01) and the Southeast Center for Agricultural Health and Injury Prevention. The research was also funded by the Department of the Army, US Army Contracting Command, Aberdeen Proving Ground, Edgewood Contracting Division, Ft Detrick MD, Deployed Warfighter Protection (DWFP) Program Grant W911QY1910003 and E911SR2210005.  The work was also supported by the Research Capacity Fund (HATCH), project award no. 02853, from the U.S. Department of Agriculture’s National Institute of Food and Agriculture. Any opinions, findings, conclusions, or recommendations expressed in this publication are those of the authors and should not be construed to represent any official USDA or U.S. Government determination or policy.”

Please include your amended statements within your cover letter; we will change the online submission form on your behalf.

4. We note that the grant information you provided in the ‘Funding Information’ and ‘Financial Disclosure’ sections do not match.

When you resubmit, please ensure that you provide the correct grant numbers for the awards you received for your study in the ‘Funding Information’ section.

5. Thank you for stating the following financial disclosure:

“The study was financially supported by the National Institutes of Health, National Institute of Allergy and Infectious Diseases (grant no. 1R03AI166406-01) and the Southeast Center for Agricultural Health and Injury Prevention. The research was also funded by the Department of the Army, US Army Contracting Command, Aberdeen Proving Ground, Edgewood Contracting Division, Ft Detrick MD, Deployed Warfighter Protection (DWFP) Program Grant W911QY1910003 and E911SR2210005.  The work was also supported by the Research Capacity Fund (HATCH), project award no. 02853, from the U.S. Department of Agriculture’s National Institute of Food and Agriculture. Any opinions, findings, conclusions, or recommendations expressed in this publication are those of the authors and should not be construed to represent any official USDA or U.S. Government determination or policy.”

Please state what role the funders took in the study.  If the funders had no role, please state: "The funders had no role in study design, data collection and analysis, decision to publish, or preparation of the manuscript."

If this statement is not correct you must amend it as needed.

Please include this amended Role of Funder statement in your cover letter; we will change the online submission form on your behalf.

6. PLOS requires an ORCID iD for the corresponding author in Editorial Manager on papers submitted after December 6th, 2016. Please ensure that you have an ORCID iD and that it is validated in Editorial Manager. To do this, go to ‘Update my Information’ (in the upper left-hand corner of the main menu), and click on the Fetch/Validate link next to the ORCID field. This will take you to the ORCID site and allow you to create a new iD or authenticate a pre-existing iD in Editorial Manager.

7. We note that Figure 1 in your submission contain map images which may be copyrighted. All PLOS content is published under the Creative Commons Attribution License (CC BY 4.0), which means that the manuscript, images, and Supporting Information files will be freely available online, and any third party is permitted to access, download, copy, distribute, and use these materials in any way, even commercially, with proper attribution. For these reasons, we cannot publish previously copyrighted maps or satellite images created using proprietary data, such as Google software (Google Maps, Street View, and Earth). For more information, see our copyright guidelines: http://journals.plos.org/plosone/s/licenses-and-copyright.

We require you to either (1) present written permission from the copyright holder to publish these figures specifically under the CC BY 4.0 license, or (2) remove the figures from your submission:

1. You may seek permission from the original copyright holder of Figure(s) [#] to publish the content specifically under the CC BY 4.0 license.

We recommend that you contact the original copyright holder with the Content Permission Form (http://journals.plos.org/plosone/s/file?id=7c09/content-permission-form.pdf) and the following text:

“I request permission for the open-access journal PLOS ONE to publish XXX under the Creative Commons Attribution License (CCAL) CC BY 4.0 (http://creativecommons.org/licenses/by/4.0/). Please be aware that this license allows unrestricted use and distribution, even commercially, by third parties. Please reply and provide explicit written permission to publish XXX under a CC BY license and complete the attached form.”

Please upload the completed Content Permission Form or other proof of granted permissions as an "Other" file with your submission.

In the figure caption of the copyrighted figure, please include the following text: “Reprinted from [ref] under a CC BY license, with permission from [name of publisher], original copyright [original copyright year].”

2. If you are unable to obtain permission from the original copyright holder to publish these figures under the CC BY 4.0 license or if the copyright holder’s requirements are incompatible with the CC BY 4.0 license, please either i) remove the figure or ii) supply a replacement figure that complies with the CC BY 4.0 license. Please check copyright information on all replacement figures and update the figure caption with source information. If applicable, please specify in the figure caption text when a figure is similar but not identical to the original image and is therefore for illustrative purposes only.

The following resources for replacing copyrighted map figures may be helpful:

USGS National Map Viewer (public domain): http://viewer.nationalmap.gov/viewer/

The Gateway to Astronaut Photography of Earth (public domain): http://eol.jsc.nasa.gov/sseop/clickmap/

Maps at the CIA (public domain): https://www.cia.gov/library/publications/the-world-factbook/index.html and https://www.cia.gov/library/publications/cia-maps-publications/index.html

NASA Earth Observatory (public domain): http://earthobservatory.nasa.gov/

Landsat: http://landsat.visibleearth.nasa.gov/

USGS EROS (Earth Resources Observatory and Science (EROS) Center) (public domain): http://eros.usgs.gov/#

Natural Earth (public domain): http://www.naturalearthdata.com/

8. If the reviewer comments include a recommendation to cite specific previously published works, please review and evaluate these publications to determine whether they are relevant and should be cited. There is no requirement to cite these works unless the editor has indicated otherwise.

[Note: HTML markup is below. Please do not edit.]

Reviewers' comments:

Reviewer's Responses to Questions

**Comments to the Author**

1. Is the manuscript technically sound, and do the data support the conclusions?

The manuscript must describe a technically sound piece of scientific research with data that supports the conclusions. Experiments must have been conducted rigorously, with appropriate controls, replication, and sample sizes. The conclusions must be drawn appropriately based on the data presented.

Reviewer #1: Partly

Reviewer #2: Yes

**********

2. Has the statistical analysis been performed appropriately and rigorously?

Reviewer #1: Yes

Reviewer #2: Yes

**********

3. Have the authors made all data underlying the findings in their manuscript fully available?

The PLOS Data policy requires authors to make all data underlying the findings described in their manuscript fully available without restriction, with rare exception (please refer to the Data Availability Statement in the manuscript PDF file). The data should be provided as part of the manuscript or its supporting information, or deposited to a public repository. For example, in addition to summary statistics, the data points behind means, medians and variance measures should be available. If there are restrictions on publicly sharing data—e.g. participant privacy or use of data from a third party—those must be specified.

Reviewer #1: No

Reviewer #2: Yes

**********

4. Is the manuscript presented in an intelligible fashion and written in standard English?

PLOS ONE does not copyedit accepted manuscripts, so the language in submitted articles must be clear, correct, and unambiguous. Any typographical or grammatical errors should be corrected at revision, so please note any specific errors here.

Reviewer #1: Yes

Reviewer #2: Yes

**********

5. Review Comments to the Author

Please use the space provided to explain your answers to the questions above. You may also include additional comments for the author, including concerns about dual publication, research ethics, or publication ethics. (Please upload your review as an attachment if it exceeds 20,000 characters)

Reviewer #1: Comments to the Author

This study investigates the bacterial microbiome of free-living chiggers in North Carolina, USA, and explores its associations with geographic factors and Orientia infection. While the research question is relevant, the manuscript contains fundamental methodological deficiencies. These deficiencies primarily concern the identification of free-living chigger specimens, the description of sampling background, and the confirmation of life-history stages.

Major Comments (must be revised or clearly explained)

1. Inadequate identification of free-living chiggers and absence of morphological evidence

The internationally accepted research framework for chiggers (Trombiculidae) is based on the examination of parasitic larvae collected directly from the body surface of animal hosts. Chiggers parasitize small mammals exclusively at the larval stage, during which they feed on host blood. After feeding, larvae detach from the host and subsequently develop into nymphs and adults. These later stages are free-living and non-parasitic. Therefore, the ecological condition of being “free-living” cannot be directly equated with chigger larvae, nor can it be used as evidence of vector competence.

(1) Absence of field recognition and morphological identification

The manuscript reports the collection of “red moving objects (<250 μm)” observed on black tiles. This description does not constitute a diagnostic characteristic of chiggers. Numerous soil microarthropods, including other mites and immature insects, may fall within this size range and display similar coloration. The authors must clarify whether voucher specimens were slide-mounted and preserved. It is also necessary to specify whether all samples, or only a subset, were examined microscopically prior to molecular analysis, including examination under oil immersion when appropriate.

Furthermore, the authors must state whether non-chigger arthropods were excluded based on fundamental larval chigger characters, such as body morphology, leg number and segmentation, and the presence and structure of the scutum. The manuscript should clearly indicate which diagnostic characters were used and which taxonomic keys or references were followed. If no morphological identification was conducted, this omission represents a serious methodological limitation. In that case, the authors must explicitly acknowledge this limitation in the Discussion and evaluate its potential impact on the study’s conclusions.

(2) Lack of confirmation of life-history stage

Only the larval stage of chiggers is parasitic and of medical relevance. The manuscript refers to the collected specimens as “free-living, unfed chiggers,” yet provides no explanation of how the authors confirmed that all specimens were larvae rather than unfed nymphs or adults. Larvae possess three pairs of legs, whereas nymphs and adults possess four pairs, a clear and diagnostic distinction. No morphological evidence is presented to confirm life-history stage. If non-larval stages were included, then the fundamental assumption regarding disease transmission potential is invalid.

2. Unclear description of DNA extraction and library preparation from single chiggers

In the section “DNA extraction and chigger identification,” the authors state that “individual, free-living chiggers were surface sterilized,” implying that DNA extraction was performed on single specimens. This raises significant technical concerns. Individual chiggers are extremely small and possess very low biomass. Obtaining sufficient DNA from a single unfed chigger larva for both high-quality 16S rRNA amplicon sequencing and 18S rRNA Sanger sequencing is technically challenging and often associated with a high failure rate.

The authors must clearly state whether each “individual” sample represents a single chigger or a pooled sample consisting of multiple chiggers collected from the same site or batch. This distinction is critical, as it determines the biological unit of downstream analyses and whether the results represent individual-level microbiomes or pooled community profiles. The authors must also report DNA extraction efficiency and PCR amplification success rates, and specify whether any samples were excluded due to insufficient DNA quantity. These details are essential for evaluating potential sample selection bias and ensuring reproducibility.

3. Inadequate description and absence of key parameters in the molecular species identification method

(1) Gene choice and similarity threshold

The use of the 18S rRNA gene for chigger species identification is acceptable. However, the manuscript does not report the sequence similarity threshold applied in BLASTn comparisons. This parameter is central to molecular taxonomic assignment and determines whether identification at the genus or species level is justified.

(2) Specific concern regarding sequence similarity

In the Results section (page 13), the manuscript reports that P. petrolinensis sequences show 95.2–96.3% similarity to reference sequences. The authors must specify the identification threshold used and justify its application. Similarity values in this range are generally insufficient for confident species-level identification and more commonly support genus-level assignment or suggest the presence of cryptic or undescribed taxa. The authors must explain why these sequences were assigned to P. petrolinensis, and clarify whether morphological evidence, similarity overlap among related taxa, or phylogenetic analyses were used to support this conclusion. The criteria for species determination must be explicitly stated.

4. Insufficient description of sampling locations and ecological background

Although several nature reserves in North Carolina are listed as sampling sites, the manuscript does not adequately describe the specific microhabitats in which mites were collected. It is also unclear whether the sampling locations were associated with small mammal activity or suitable host habitats, or whether potential hosts were present in the vicinity.

Under the current description, the collection of free-moving mites from surface environments alone is insufficient to establish that the specimens are chigger larvae with prior parasitic history or vector potential.

Recommendations for Revision

If the identity of the study organisms as chigger larvae has not been reliably confirmed, then all subsequent inferences are based on an unstable biological premise. The manuscript therefore exhibits a high risk of over-interpretation. In a revised version, the authors must address at least the following points:

1. Provide systematic morphological identification evidence and clearly confirm the life-history stage of all specimens.

2. Explicitly state the similarity thresholds and decision criteria used for 18S rRNA-based molecular identification.

3. In the DNA extraction section, clearly define what constitutes a single sample—whether it is an individual chigger mite or a pooled sample of chigger mites. Include detailed extraction procedures, as well as the success rates of extraction and PCR.

4. If larval identity cannot be confirmed, use the term “chiggers” with caution throughout the manuscript and fully discuss this limitation.

Reviewer #2: The manuscript "Geographic and Orientia infection status influence on the bacterial microbiome of freeliving chiggers in North Carolina, USA" describes sampling of 3 different species of chiggers from 3 different ecological region in the state of North Carolina in the US, and a subsequent characterization of their bacterial microbiome using 16S amplicon sequencing. Fleas and chiggers are historically known vectors of various infectious diseases, but are also emerging as potential vectors of new pathogens in new geographical areas. Characterizing their geographical distribution as well as their community of microbes is important for both ecological studies as well as public health reasons. This well-written manuscript is a welcome addition to this area of research. The overall experimental design is appropriate, experiments are well executed, and data analysis and presentation is of good quality. The details of experimental methods and data analysis is described in reasonable detail. The raw data seem to have been deposited in the public NCBI SRA database under bioproject PRJNA1285095 and SUB15424311. which will hopefully be released publicly upon acceptance/publication of the manuscript.

There are only 3 major a few minor points that the authors need to address before this manuscript can be accepted for publication.

Major points:

1. In the methods section, the authors have not specified the Illumina instrument used for sequencing. This is critical information, as it influences the choice of denoising algorithm. Starting with NovaSeq instruments, Illumina started binning per-base quality scores instead of a monotonic continuous scale. However, the DADA2 pipeline assumes a continuous scoring scheme and is therefore not suitable for FASTQs produced by NovaSeq or later instruments. If the authors used an Illumina instrument which compresses FASTQ quality scores into 4 or 5 bins (e.g. NovaSeq), DADA2 cannot be used and Deblur is recommended instead. For more discussions see https://github.com/benjjneb/dada2/issues/791 and https://forum.qiime2.org/t/novaseq-and-dada2-incompatibility/25865 . Please provide instrument details and choose Deblur over DADA2 if needed.

2. The Greengenes database has been superseded by Greengenes2 for quite some time now, with obvious improvements in taxonomical breadth and accuracy. As per their methods, the authors have used "Greengenes 13_8" which was last updated in 2013 (https://ftp.microbio.me/greengenes_release/). Please use the latest GreenGenes2 database in all analysis. For more discussion see https://forum.qiime2.org/t/greengenes2-2024-09/31606. Also, update the citation 40 from GreenGenes to Greengenes2, described in McDonald et al. Nature Biotechnology (2023) : https://www.nature.com/articles/s41587-023-01845-1

3. In Results, starting line 248 onwards, the authors have described the taxa observed in the subset ASVs that *could* be classified ("32 bacterial phyla, 87 classes, 158 orders, 282 families, and 521 genera"). However, it is not clear how many of the 3,575 ASVs could be classified into these levels versus what fraction of ASVs remained unclassified. Please provide these proportions at all taxonomic levels. It is not uncommon for more than 70% of the ASVs to remain unclassified, especially for under-studied microbiomes such the one these authors are studying - but discussing only the classifiable fraction creates to a false impression that there is no more bacterial diversity remaining to be discovered in these microbiomes!

Minor points:

1. Median values are a more robust statistical descriptors of distribution of data than means. Hence it is recommended that the authors report median values wherever possible e.g. line 246 "average of 94,989 reads per sample", line 351 onwards multiple instances of "mean relative abundance".

2. Line 247: "After filtering ASVs based on total abundance and prevalence in the

dataset" : What were the criteria/cut-offs used in this filtering step?

3. Line 460 onwards in Discussion: The authors find the absence of difference in alpha diversity measures between different host chigger species. However, alpha diversity is a within sample measurement. Thus it is technically possible for two microbiomes to be composed of different species (represented by different unique ASV sequences) but have the same diversity metrics (same *number* of unique ASVs). Here is a very simplified toy example to show this: chigger-species1 has 3 microbes A, B and C while chigger-species2 has three different microbes D, E, and F - such that the diversity metric is 3 for both chigger-species.

Did the authors check if indeed the same set of ASVs were found in all three chigger species? Please also report the median number of ASVs observed per sample (not all 3,575 ASVs are expected to be seen in each sample).

3. In Figures 2 and 3, "Observed fatures" could be changed to "Observed ASVs"?

4. The reference 30 cites a PhD thesis, which I believe has now been published as a peer-reviewed research article: Alkathiry et al. Microbiome and mitogenomics of the chigger mite Pentidionis agamae: potential role as an Orientia vector and associations with divergent clades of Wolbachia and Borrelia. BMC Genomics (2024) at https://doi.org/10.1186/s12864-024-10301-6 . Please update accordingly.

*-*-*

**********

6. PLOS authors have the option to publish the peer review history of their article (what does this mean?). If published, this will include your full peer review and any attached files.

If you choose “no”, your identity will remain anonymous but your review may still be made public.

**Do you want your identity to be public for this peer review?**  For information about this choice, including consent withdrawal, please see our Privacy Policy.

Reviewer #1: No

Reviewer #2: No

**********

[NOTE: If reviewer comments were submitted as an attachment file, they will be attached to this email and accessible via the submission site. Please log into your account, locate the manuscript record, and check for the action link "View Attachments". If this link does not appear, there are no attachment files.]

To ensure your figures meet our technical requirements, please review our figure guidelines: https://journals.plos.org/plosone/s/figures

You may also use PLOS’s free figure tool, NAAS, to help you prepare publication quality figures: https://journals.plos.org/plosone/s/figures#loc-tools-for-figure-preparation.

NAAS will assess whether your figures meet our technical requirements by comparing each figure against our figure specifications.

Attachment Submitted filename: Comments to the Author.pdf

# Author comment: Author response to Decision Letter 1

**DOI:** 10.1371/journal.pone.0353174.r002

20 May 2026

Reviewer #1: Comments to the Author

This study investigates the bacterial microbiome of free-living chiggers in North Carolina, USA, and explores its associations with geographic factors and Orientia infection. While the research question is relevant, the manuscript contains fundamental methodological deficiencies. These deficiencies primarily concern the identification of free-living chigger specimens, the description of sampling background, and the confirmation of life-history stages.

Authors’ response: We thank the reviewer for the careful evaluation of our manuscript and for recognizing the relevance of the research question, particularly regarding chigger specimen identification and confirmation of life-history stages. Detailed replies to the comments are given below.

Major Comments (must be revised or clearly explained)

1. Inadequate identification of free-living chiggers and absence of morphological evidence

The internationally accepted research framework for chiggers (Trombiculidae) is based on the examination of parasitic larvae collected directly from the body surface of animal hosts. Chiggers parasitize small mammals exclusively at the larval stage, during which they feed on host blood. After feeding, larvae detach from the host and subsequently develop into nymphs and adults. These later stages are free-living and non-parasitic. Therefore, the ecological condition of being “free-living” cannot be directly equated with chigger larvae, nor can it be used as evidence of vector competence.

Authors’ response: We appreciate the reviewer’s critical distinction between the parasitic larval stage and the free-living post-larval stages (nymphs and adults) of Trombiculidae, and we agree that vector competence is exclusive to the larvae. To clarify, our use of the term “free-living” referred specifically to the collection site (unattached larvae questing in soil and vegetation) rather than their biological stage. To address the reviewer’s concern regarding identification and morphological evidence, all collected specimens were examined under a stereo microscope to confirm they were in the hexapod (six-legged) larval stage, distinguishing them from the octopod (eight-legged) nymphs and adults. Furthermore, to ensure taxonomic accuracy, representative sub-samples from these collections were validated by Dr. Dac Crossley (Curator of Acari at the Georgia Museum of Natural History, Georgia, USA) a specialist in Trombiculid taxonomy, to ensure the stage and species-level identification. We have updated the Methods section to explicitly state these morphological criteria and our external validation process.

(1) Absence of field recognition and morphological identification

The manuscript reports the collection of “red moving objects (<250 μm)” observed on black tiles. This description does not constitute a diagnostic characteristic of chiggers. Numerous soil microarthropods, including other mites and immature insects, may fall within this size range and display similar coloration. The authors must clarify whether voucher specimens were slide-mounted and preserved. It is also necessary to specify whether all samples, or only a subset, were examined microscopically prior to molecular analysis, including examination under oil immersion when appropriate.

Furthermore, the authors must state whether non-chigger arthropods were excluded based on fundamental larval chigger characters, such as body morphology, leg number and segmentation, and the presence and structure of the scutum. The manuscript should clearly indicate which diagnostic characters were used and which taxonomic keys or references were followed. If no morphological identification was conducted, this omission represents a serious methodological limitation. In that case, the authors must explicitly acknowledge this limitation in the Discussion and evaluate its potential impact on the study’s conclusions.

Authors’ Response: We agree with the reviewer that the field observation of "red moving objects (<250 μm)" on black tiles is not diagnostic of chiggers and could apply to various soil microarthropods. This description was intended only to reflect the initial field observation and was not the primary basis for identification.

Following collection, specimens were transported to the laboratory and examined under a stereomicroscope. Identification as larval chiggers (family Trombiculidae) was confirmed based on established diagnostic criteria, including, the presence of six legs (distinguishing larvae from nymphal and adult mites), overall body morphology consistent with trombiculid larvae, and characteristic leg segmentation as described in taxonomic keys (Wharton et al., 1952; Brennan et al., 1959; Bennett et al., 2014). These features were used to exclude non-chigger arthropods collected during environmental sampling.

To ensure accuracy of life stage and species identification, randomly selected subsamples from each collection site were submitted to Dr. Dac Crossley, Curator of Acari at the Georgia Museum of Natural History, for independent life-stage and morphological validation. Representative specimens from each collection batch were slide-mounted, preserved as vouchers, and examined via compound microscopy (including oil-immersion where necessary). Identification was performed using standard taxonomic keys (Wharton et al., 1952; Brennan et al., 1959; Bennett et al., 2014).

Because slide-mounting permanently alters specimens, detailed morphological examination was conducted on representative subsamples rather than the entire collection. All remaining specimens were subsequently validated using molecular methods prior to downstream analyses. The Methods section has been revised to explicitly detail the morphological examination procedures, the subsampling strategy, and the external taxonomic validation.

(2) Lack of confirmation of life-history stage

Only the larval stage of chiggers is parasitic and of medical relevance. The manuscript refers to the collected specimens as “free-living, unfed chiggers,” yet provides no explanation of how the authors confirmed that all specimens were larvae rather than unfed nymphs or adults. Larvae possess three pairs of legs, whereas nymphs and adults possess four pairs, a clear and diagnostic distinction. No morphological evidence is presented to confirm life-history stage. If non-larval stages were included, then the fundamental assumption regarding disease transmission potential is invalid.

Authors’ response: As we stated in our response above, the collected samples were examined after collection to confirm that they were in the larval stage of mites. We acknowledge that these essential identification steps were not described in sufficient detail in the original manuscript, which may have caused confusion regarding how the life-history stage was verified. This level of “due diligence” to identify specimens is standard practice for our group and applied also to our previously published papers on our work with chiggers in the US and their infection with Orientia spp. bacteria.

2. Unclear description of DNA extraction and library preparation from single chiggers

In the section “DNA extraction and chigger identification,” the authors state that “individual, free-living chiggers were surface sterilized,” implying that DNA extraction was performed on single specimens. This raises significant technical concerns. Individual chiggers are extremely small and possess very low biomass. Obtaining sufficient DNA from a single unfed chigger larva for both high-quality 16S rRNA amplicon sequencing and 18S rRNA Sanger sequencing is technically challenging and often associated with a high failure rate. The authors must clearly state whether each “individual” sample represents a single chigger or a pooled sample consisting of multiple chiggers collected from the same site or batch. This distinction is critical, as it determines the biological unit of downstream analyses and whether the results represent individual-level microbiomes or pooled community profiles. The authors must also report DNA extraction efficiency and PCR amplification success rates, and specify whether any samples were excluded due to insufficient DNA quantity. These details are essential for evaluating potential sample selection bias and ensuring reproducibility.

Authors’ response: We confirm that DNA extraction was performed on individual chigger larvae; each “individual” sample reported in the manuscript represents a single chigger rather than a pooled specimen. Initially, 120 chiggers were processed separately for DNA extraction. To maximize DNA recovery and concentration from these single specimens, we optimized the extraction protocol by reducing the final elution volume to 50 μL (instead of the manufacturer’s recommended 200 μL). This modification ensured sufficient DNA concentration for downstream PCR applications. Furthermore, advances in molecular technology now allow for the detection of DNA from extremely small biological samples, even from a single hair, supporting the feasibility and reliability of single-specimen DNA analysis.

Following extraction, NanoDrop spectrophotometry was used as an initial quality control step. Twenty-five samples did not yield detectable DNA and were therefore excluded from further analysis. The remaining 95 individual samples were subjected to 16S rRNA microbiome analysis, as described in Chen et al. (2023). As noted in Chen et al. (2023), chigger species identification was performed using both morphological and molecular approaches on subsampled material. For the present study, we conducted 18S rRNA gene amplification and Sanger sequencing on all 95 samples to enable species-level identification and to assess microbiome variation among species. Although 18S amplification was successful for all samples, 26 produced ambiguous sequencing results. Consequently, 69 samples yielded high-quality sequences suitable for identification, while the 26 ambiguous samples were excluded from the final analysis. These methodological details and the technical limitations inherent to single-chigger DNA extraction have now been clarified and incorporated into the revised manuscript.

3. Inadequate description and absence of key parameters in the molecular species identification method

(1) Gene choice and similarity threshold

The use of the 18S rRNA gene for chigger species identification is acceptable. However, the manuscript does not report the sequence similarity threshold applied in BLASTn comparisons. This parameter is central to molecular taxonomic assignment and determines whether identification at the genus or species level is justified.

Authors’ response: We agree that this was not explained in the earlier version of the manuscript. In the revised manuscript, we have now explicitly described the criteria applied for BLASTn-based identification using the 18S rRNA gene.

(2) Specific concern regarding sequence similarity

In the Results section (page 13), the manuscript reports that P. petrolinensis sequences show 95.2–96.3% similarity to reference sequences. The authors must specify the identification threshold used and justify its application. Similarity values in this range are generally insufficient for confident species-level identification and more commonly support genus-level assignment or suggest the presence of cryptic or undescribed taxa. The authors must explain why these sequences were assigned to P. petrolinensis, and clarify whether morphological evidence, similarity overlap among related taxa, or phylogenetic analyses were used to support this conclusion. The criteria for species determination must be explicitly stated.

Authors’ response: We acknowledge that the observed sequence similarity of 95.2–96.3% falls below the commonly accepted 97–99% threshold for confident species-level identification in many taxa. Therefore, we have revised the classification to the genus Pseudoschoengastia, consistent with the supporting morphological evidence. The manuscript has been updated accordingly, and the rationale for this change is now clearly explained in the revised version.

4. Insufficient description of sampling locations and ecological background

Although several nature reserves in North Carolina are listed as sampling sites, the manuscript does not adequately describe the specific microhabitats in which mites were collected. It is also unclear whether the sampling locations were associated with small mammal activity or suitable host habitats, or whether potential hosts were present in the vicinity. Under the current description, the collection of free-moving mites from surface environments alone is insufficient to establish that the specimens are chigger larvae with prior parasitic history or vector potential.

Authors’ response: We sincerely thank the reviewer for this valuable comment. Unfortunately, detailed ecological data were not collected at the time of sampling. Specifically, we did not record microhabitat characteristics, small mammal activity, host presence, or vegetation type at the collection sites. In addition, chiggers were collected exclusively from surface environments and were not obtained directly from vertebrate hosts.

Recommendations for Revision

If the identity of the study organisms as chigger larvae has not been reliably confirmed, then all subsequent inferences are based on an unstable biological premise. The manuscript therefore exhibits a high risk of over-interpretation. In a revised version, the authors must address at least the following points:

1. Provide systematic morphological identification evidence and clearly confirm the life-history stage of all specimens.

Authors’ response: We confirm that all specimens were subjected to systematic morphological examination using standard taxonomic keys and diagnostic characters specific to this group. The distinguishing morphological features used for identification are now clearly described in the revised manuscript to ensure transparency in taxonomic assignment. All specimens analyzed in this study were confirmed to be in the larval stage based on characteristic morphological traits (e.g., number of legs, body segmentation). This clarification has been added to the Methods section to eliminate any ambiguity regarding the life-history stage of the analyzed specimens.

2. Explicitly state the similarity thresholds and decision criteria used for 18S rRNA-based molecular identification.

Authors’ response: We agree that the decision criteria for 18S rRNA–based identification should be explicitly stated. This concern has been addressed in the revised manuscript, where we now detail the thresholds and the decision framework used for taxonomic assignments.

3. In the DNA extraction section, clearly define what constitutes a single sample—whether it is an individual chigger mite or a pooled sample of chigger mites. Include detailed extraction procedures, as well as the success rates of extraction and PCR.

Authors’ response: We can confirm that each sample consisted of a single, individual chigger mite rather than a pooled sample. As the reviewer’s suggested, we have also added a detailed step-by-step extraction procedure and included the specific success rates for both the DNA extraction and the subsequent PCR amplification.

4. If larval identity cannot be confirmed, use the term “chiggers” with caution throughout the manuscript and fully discuss this limitation.

Authors’ response: In the revised manuscript, we have clarified in the methodology that the larval identity was confirmed as chiggers based on the presence of six legs. Because our morphological identification was definitive, we believe the term 'chiggers' is used correctly. However, as requested, we have added a brief statement in the Methodology and Discussion sections to provide the specific morphological criteria used for this confirmation.

Reviewer #2:

The manuscript "Geographic and Orientia infection status influence on the bacterial microbiome of freeliving chiggers in North Carolina, USA" describes sampling of 3 different species of chiggers from 3 different ecological region in the state of North Carolina in the US, and a subsequent characterization of their bacterial microbiome using

Attachment Submitted filename: Comments and response to Reviewer #1and2_Final.docx

# Peer review: Decision Letter 1

**DOI:** 10.1371/journal.pone.0353174.r003

**Authors:** Yong Qi

21 Jun 2026

Geographic and Orientia infection status influence on the bacterial microbiome of free-living chiggers in North Carolina, USA

PONE-D-25-67871R1

Dear Dr. Ponnusamy,

We’re pleased to inform you that your manuscript has been judged scientifically suitable for publication and will be formally accepted for publication once it meets all outstanding technical requirements.

Within one week, you’ll receive an e-mail detailing the required amendments. When these have been addressed, you’ll receive a formal acceptance letter and your manuscript will be scheduled for publication.

An invoice will be generated when your article is formally accepted. Please note, if your institution has a publishing partnership with PLOS and your article meets the relevant criteria, all or part of your publication costs will be covered. Please make sure your user information is up-to-date by logging into Editorial Manager at Editorial Manager® and clicking the ‘Update My Information' link at the top of the page. For questions related to billing, please contact billing support.

If your institution or institutions have a press office, please notify them about your upcoming paper to help maximize its impact. If they’ll be preparing press materials, please inform our press team as soon as possible -- no later than 48 hours after receiving the formal acceptance. Your manuscript will remain under strict press embargo until 2 pm Eastern Time on the date of publication. For more information, please contact onepress@plos.org.

Kind regards,

Yong Qi

Academic Editor

PLOS One

Additional Editor Comments (optional):

Reviewers' comments:

Reviewer's Responses to Questions

**Comments to the Author**

1. If the authors have adequately addressed your comments raised in a previous round of review and you feel that this manuscript is now acceptable for publication, you may indicate that here to bypass the “Comments to the Author” section, enter your conflict of interest statement in the “Confidential to Editor” section, and submit your "Accept" recommendation.

Reviewer #1: All comments have been addressed

Reviewer #2: All comments have been addressed

**********

2. Is the manuscript technically sound, and do the data support the conclusions?

The manuscript must describe a technically sound piece of scientific research with data that supports the conclusions. Experiments must have been conducted rigorously, with appropriate controls, replication, and sample sizes. The conclusions must be drawn appropriately based on the data presented.

Reviewer #1: Partly

Reviewer #2: Yes

**********

3. Has the statistical analysis been performed appropriately and rigorously?

Reviewer #1: Yes

Reviewer #2: Yes

**********

4. Have the authors made all data underlying the findings in their manuscript fully available?

The PLOS Data policy requires authors to make all data underlying the findings described in their manuscript fully available without restriction, with rare exception (please refer to the Data Availability Statement in the manuscript PDF file). The data should be provided as part of the manuscript or its supporting information, or deposited to a public repository. For example, in addition to summary statistics, the data points behind means, medians and variance measures should be available. If there are restrictions on publicly sharing data—e.g. participant privacy or use of data from a third party—those must be specified.

Reviewer #1: No

Reviewer #2: Yes

**********

5. Is the manuscript presented in an intelligible fashion and written in standard English?

PLOS ONE does not copyedit accepted manuscripts, so the language in submitted articles must be clear, correct, and unambiguous. Any typographical or grammatical errors should be corrected at revision, so please note any specific errors here.

Reviewer #1: Yes

Reviewer #2: Yes

**********

6. Review Comments to the Author

Please use the space provided to explain your answers to the questions above. You may also include additional comments for the author, including concerns about dual publication, research ethics, or publication ethics. (Please upload your review as an attachment if it exceeds 20,000 characters)

Reviewer #1: I appreciate the authors’ comprehensive and thoughtful responses to the peer review feedback. The revised manuscript reflects a diligent effort to incorporate the suggested changes, which has markedly elevated the overall quality, rigor, and clarity of the paper. The authors' constructive and meticulous attitude throughout this revision process is highly commendable.

It is worth noting that the initial submission exhibited certain fundamental limitations—challenges that are often difficult to entirely circumvent given the constraints of the study design and the nature of the data. However, the authors have addressed these underlying issues as thoroughly and transparently as practically possible. While some of these inherent methodological constraints remain largely unavoidable, the revisions, alongside the carefully expanded discussion of these limitations, have satisfactorily mitigated their impact on the study's core conclusions.

In light of these substantial improvements, I am largely satisfied with the current iteration of the manuscript and believe the authors have adequately resolved the primary concerns raised during the initial review. The manuscript now aligns well with the scientific standards expected for publication in PLOS One.

Reviewer #2: (No Response)

**********

7. PLOS authors have the option to publish the peer review history of their article (what does this mean?). If published, this will include your full peer review and any attached files.

If you choose “no”, your identity will remain anonymous but your review may still be made public.

**Do you want your identity to be public for this peer review?**  For information about this choice, including consent withdrawal, please see our Privacy Policy.

Reviewer #1: No

Reviewer #2: No

**********

# Editor report: Acceptance letter

**DOI:** 10.1371/journal.pone.0353174.r004

**Authors:** Yong Qi

PONE-D-25-67871R1

PLOS One

Dear Dr. Ponnusamy,

I'm pleased to inform you that your manuscript has been deemed suitable for publication in PLOS One. Congratulations! Your manuscript is now being handed over to our production team.

At this stage, our production department will prepare your paper for publication. This includes ensuring the following:

* All references, tables, and figures are properly cited

* All relevant supporting information is included in the manuscript submission,

* There are no issues that prevent the paper from being properly typeset

You will receive further instructions from the production team, including instructions on how to review your proof when it is ready. Please keep in mind that we are working through a large volume of accepted articles, so please give us a few days to review your paper and let you know the next and final steps.

Lastly, if your institution or institutions have a press office, please let them know about your upcoming paper now to help maximize its impact. If they'll be preparing press materials, please inform our press team within the next 48 hours. Your manuscript will remain under strict press embargo until 2 pm Eastern Time on the date of publication. For more information, please contact onepress@plos.org.

You will receive an invoice from PLOS for your publication fee after your manuscript has reached the completed accept phase. If you receive an email requesting payment before acceptance or for any other service, this may be a phishing scheme. Learn how to identify phishing emails and protect your accounts at https://explore.plos.org/phishing.

If we can help with anything else, please email us at customercare@plos.org.

Thank you for submitting your work to PLOS ONE and supporting open access.

Kind regards,

PLOS ONE Editorial Office Staff

on behalf of

Dr. Yong Qi

Academic Editor

PLOS One
